# Single-cell RNA sequencing reveals a pro-invasive cancer-associated fibroblast subgroup associated with poor clinical outcomes in patients with gastric cancer

**DOI:** 10.7150/thno.60540

**Published:** 2022-01-01

**Authors:** Xuechun Li, Zhao Sun, Gongxin Peng, Yi Xiao, Junchao Guo, Bin Wu, Xiaoyi Li, Weixun Zhou, Jiarui Li, Zhe Li, Chunmei Bai, Lin Zhao, Qin Han, Robert Chunhua Zhao, Xiaoyue Wang

**Affiliations:** 1 Institute of Basic Medical Sciences Chinese Academy of Medical Sciences, School of Basic Medicine Peking Union Medical College, Peking Union Medical College Hospital, Center of Excellence in Tissue Engineering Chinese Academy of Medical Sciences, Beijing Key Laboratory (No. BZO381), Beijing, People's Republic of China.; 2 Department of Medical Oncology, Peking Union Medical College Hospital, Chinese Academy of Medical Sciences, Beijing, China.; 3 Center for Bioinformatics, Institute of Basic Medical Sciences, Chinese Academy of Medical Sciences, School of Basic Medicine, Peking Union Medical College, Beijing, 100005, China.; 4 Department of General Surgery, Peking Union Medical College Hospital, Chinese Academy of Medical Sciences, Beijing, China; 5 Department of Pathology, Peking Union Medical College Hospital, Chinese Academy of Medical Sciences, Beijing, China; 6 Department of Gynecologic Oncology, National Cancer Center/ National Clinical Research Center for Cancer/Cancer Hospital, Chinese Academy of Medical Sciences and Peking Union Medical College, Beijing, China; 7 School of Life Sciences, Shanghai University, 99 Shangda Road, Shanghai 200444, China

**Keywords:** Gastric cancer, Tumor microenvironment, CAF heterogeneity, iCAF, eCAF, scRNA-Seq, Multistaining registration

## Abstract

**Background:** The protumor activities of cancer-associated fibroblasts (CAFs) suggest that they are potential therapeutic targets for the treatment of cancer. The mechanism of CAF heterogeneity in gastric cancer (GC) remains unclear and has slowed translational advances in targeting CAFs. Therefore, a comprehensive understanding of the classification, function, activation stage, and spatial distribution of the CAF subsets in GC is urgently needed.

**Methods:** In this study, the characteristics of the CAF subsets and the dynamic communication among the tumor microenvironment (TME) components regulated by the CAF subsets were analyzed by performing single-cell RNA sequencing of eight pairs of GC and adjacent mucosal (AM) samples. The spatial distribution of the CAF subsets in different Lauren subtypes of GC, as well as the neighborhood relations between these CAF subsets and the protumor immune cell subsets were evaluated by performing multistaining registration.

**Results:** Tumor epithelial cells exhibited significant intratumor and intertumor variabilities, while CAFs mainly exhibited intratumor variability. Moreover, we identified four CAF subsets with different properties in GC. These four CAF subsets shared similar properties with their resident fibroblast counterparts in the adjacent mucosa but also exhibited enhanced protumor activities. Additionally, two CAF subsets, inflammatory CAFs (iCAFs) and extracellular matrix CAFs (eCAFs), communicated with adjacent immune cell subsets in the GC TME. iCAFs interacted with T cells by secreting interleukin (IL)-6 and C-X-C motif chemokine ligand 12 (CXCL12), while eCAFs correlated with M2 macrophages via the expression of periostin (POSTN). eCAFs, which function as a pro-invasive CAF subset, decreased the overall survival time of patients with GC.

**Conclusions:** iCAFs and eCAFs not only exhibited enhanced pro-invasive activities but also mobilized the surrounding immune cells to construct a tumor-favorable microenvironment. Therefore, inhibiting their activation restrains the GC 'seed' and simultaneously improves the 'GC' soil, suggesting that it represents a promising therapeutic strategy for the treatment of GC.

## Background

According to Global Cancer Statistics 2020, gastric cancer (GC) is one of the most frequently diagnosed cancers in both sexes and is the fourth leading cause of cancer-related death worldwide 1. Patients with advanced GC exhibit a poor prognosis, with a median survival of less than one year 2. Therefore, the development of novel drugs is urgently needed to improve the overall survival rates of patients with GC. Although immunotherapy is a treatment option for specific subtypes of GC, the heterogeneity of GC remains a critical barrier to the development of effective drugs that improve the prognosis of patients with GC 3.

Histopathologically, GC is classified into intestinal, diffuse, and mixed-type GC 4. Intestinal-type GC, which often evolves sequentially via chronic gastritis, atrophy, intestinal metaplasia, and dysplasia, is characterized by the glandular appearance of cells. In contrast, more aggressive diffuse-type GC is characterized by a lack of intercellular adhesion and poor prognosis; however, the molecular mechanism underlying the poor prognosis of patients with diffuse-type GC remains ambiguous 5. Several genomic studies have documented subtype-specific genetic and epigenetic alterations in GCs. The molecular heterogeneity of GCs has been reported to be associated with tumor progression and the treatment outcomes of patients 6.

Single-cell RNA sequencing (scRNA-seq) has recently been used to determine the cellular and molecular heterogeneity of GC samples 7. A recent scRNA-seq study revealed that the expression patterns of differentiation genes differ among the malignant cells of distinct pathological subtypes of GC 8. Cellular heterogeneity is also observed in the gastric mucosa of premalignant lesions preceding intestinal-type GC 9. In addition to the heterogeneity of malignant cells, scRNA-seq has also revealed transcriptional heterogeneity and widespread reprogramming of cells in the tumor microenvironment (TME), particularly immune cells in the GC TME 10.

However, the underlying mechanisms of action of endothelial cells and cancer-associated fibroblasts (CAFs) in GC, which play critical roles in the progression and metastasis of GC, remain unclear. Endothelial cells and CAFs induce tumor progression and metastasis by regulating angiogenesis, organization of the extracellular matrix (ECM), and inflammation in the TME 11. A new generation of drugs targeting the tumor stroma in the TME, such as angiogenesis inhibitors and ECM normalization drugs, are currently being tested for therapeutic use 12, 13. However, these drugs exhibit limited efficacies in a small percentage of patients. Therefore, a more comprehensive profile of stromal cells in the TME and a deeper understanding of the relationship between the different pathological subtypes of GC and TME cell subgroups are urgently required.

Here, we aimed to study the heterogeneity of stromal cells in the GC TME using scRNA-seq. By analyzing cells from advanced gastric tumors and matching them to the adjacent gastric mucosal tissues at the single-cell level, we profiled a TME map of GC and identified different stromal cell subsets that were characterized by different functions in tumor progression. Furthermore, we evaluated the spatial distribution of the significant cell subsets using immunohistochemical (IHC) staining.

## Methods

### Human Tumor Specimens

All patients with gastric cancer who enrolled in this study at the Peking Union Medical College Hospital (PUMCH) provided consent. This study was approved by the Ethics Committee of PUMCH (ethical approval number: ZS-2087). The age and sex of the patients included in this study are listed in Table S1. None of the patients received any type of neoadjuvant treatment prior to tissue collection. Fresh specimens of tumor samples and adjacent mucosal samples were resected during surgery. We ensured that fresh adjacent specimens of normal tissues located more than 5 cm from the edge of the tumor were collected. Five centimeters is the accepted standard for the collection of adjacent normal tissue specimens, and the surgeon determined that it was not a tumor sample. The histopathologist confirmed that no cancer remained in the cut ends of the resected samples. After resection, the tumors and nonmalignant tissues were separately digested into single-cell suspensions and profiled using droplet-based scRNA-seq.

### Tissue Dissociation

Fresh biopsy samples of gastric cancer tissues were cut into pieces and washed with phosphate-buffered saline (PBS; Gibco™, USA) before being dissociated with a Human Tumor Dissociation Kit (Miltenyi Biotec, Bergisch Gladbach, Germany). The whole tissue dissociation process was performed according to the protocol provided with the kit. The sample was incubated for 30 min at 37 °C until no tissue pieces were visible. Dissociated cells were pelleted by centrifugation at 1200 rpm for 5 min and resuspended in PBS. The cells were then treated with RBC lysis buffer (no. R1010; Solarbio, China) for 15 min on ice and washed once with PBS before counting. Cell viability was determined by staining the samples with 0.4% trypan blue (no. C0040; Solarbio, China).

### Droplet-based single-cell RNA sequencing

The dissociated cells were sorted into Dulbecco's PBS + 0.04% bovine serum albumin (BSA; Yeasen, China) and incubated on ice before counting. Sorted cells were then assessed for viability by staining them with Trypan blue before counting using a Countess II automated counter (Thermo Fisher Scientific, USA). Single-cell suspensions were converted to barcoded scRNA-seq libraries using the Chromium Single Cell Chip Kit (10x Genomics, USA) along with the reverse transcription (RT) master mix and single cell 3 gel beads according to the manufacturer's protocol, with the aim of estimating 6,000 cells per library. Sequencing libraries were generated using a unique sample index for each sample. Subsequently, the libraries were sequenced using an Illumina HiSeq4000 sequencer (Illumina, USA).

### Single cell RNA-seq data analysis

scRNA-seq data were processed and quantified using the Cell Ranger (2.0.1) pipeline (https://support.10xgenomics.com/single-cell-gene-expression/software/pipelines/latest/using/count) with “--id --transcriptome --fastqs --localcores' arguments. First, the hg19 reference used to align the reads was obtained from 10x genomics. The sequenced FASTQ files of 16 tissues (cancer and adjacent mucosa) for eight samples were then aligned to the hg19 human reference genome using STAR software and the Cell Ranger 'count' module. A feature-barcode matrix was generated, and the cancer and normal samples were aggregated using the Cell Ranger 'aggr' module for downstream analyses. Cells with fewer than 400 expressed genes, as well as genes expressed in less than four cells, were removed. We used the R package Seurat 3.1 to correct the batch effects before conducting the combined analysis of the scRNA-seq datasets of the eight pairs of tumor and adjacent mucosal samples.

### Clustering and marker gene identification

Normalization, clustering, differential gene expression analyses, and visualization were performed using the R package Seurat 3.1. Clustering of cells was performed using the implemented community identification method in the 'FindClusters' function and visualized using the RunUMAP function. The specific marker genes of cell clusters were identified using the Seurat 'FindMakers' function. Specifically, the differentially expressed genes (DEGs) for a specific cluster were identified by comparing cells from that cluster to all other cells using the Wilcoxon rank-sum test. Bonferroni-corrected p values less than 0.05 were used as cutoffs for identifying statistically significant DEGs. Marker genes were selected as the genes with average expression in the cluster that was more than 2-fold higher than their expression in other clusters. The canonical marker genes and top-ranked differentially expressed genes were used to annotate the cell types for each cluster. We used the 'CellCycleScoring' function in Seurat to identify cell cycle phase-specific changes in different cell clusters. The 'CellCycleScoring' function assigns each cell a score based on the expression of G2/M and S phase markers. The G2/M or S phase scores were inversely correlated, and cells that did not express G2/M and S phase markers were in G1 phase. The 'CellCycleScoring' function will assign each cell a predicted classification based on its score.

### Analysis of single-cell trajectories

Single-cell trajectories were built using Monocle2 (R package) based on the results obtained with Seurat (R package). Genes included in the analysis were selected by applying the following criteria: (1) expressed in more than 4 cells, (2) the average expression level was greater than 0.1, and (3) the q-value was less than 0.01 in differentially expressed gene expression analysis.

### Gene Ontology (GO) analysis

The GO enrichment analysis was performed on the marker genes (P values were adjusted by the Bonferroni correction < 0.01) in each cluster using Metascape.org 14. Tumor progression-associated GO terms were selected for heatmap construction from terms with P < 0.05.

### Partial epithelial-to-mesenchymal transition (p-EMT) signature scores

The 97 p-EMT genes (without *TNFRSF6B*, *CXCR7*, and *ANXA8L1* genes) based on the list reported by Sidharth et al. were used to calculate the EMT signature scores of epithelial cells 15. The p-EMT meta-signature scores for each cell were calculated as the mean expression rank for the 97 genes based on their expression level (RPKM) in all cells. The nonparametric Wilcoxon rank sum test was performed to assess differences in p-EMT meta-signature scores among the different groups.

### The Cancer Genome Atlas (TCGA) data analysis

Bulk RNA-seq count data from 377 patients with gastric cancer were downloaded using the Bioconductor TCGA biolinks package (version 2.14.0). The count data were converted to transcripts per million (TPM) using the 'countToTpm_matrix' function in the R package 'GeoTcgaData'. The expression levels of each marker gene were normalized among 377 patients by calculating Z scores. Clinical data and immune cell fraction data were downloaded from the supplementary data in the study by Thorsson et al. 16. The correlations between marker gene expression and the relative abundance of different immune cell types were calculated using the Spearman method. The correlation of each marker gene with overall survival was calculated using a Cox proportional hazards model (coxph in R survival package version), including age, sex, tumor stage, and the mean expression of marker genes as variables, and the significance value was calculated using the Wald test. For plotting the Kaplan-Meier plots, marker gene expression was categorized as high if it was within the top 30% of all samples.

### Immunofluorescence staining

After deparaffinization and rehydration, antigen retrieval was performed in EDTA antigen repair solution (pH 8.0) (Servicebio, China. No. G1206) using a microwave oven. Tissue sections were first boiled using medium power for 8 min, using medium power for 8 min, and then medium-low power for 7 min. After three washes with PBS (Servicebio, China. No. G0002), tissue sections were blocked in 3% BSA. Tissue sections were then incubated with primary antibodies (von Willebrand factor (VWF), 1:1000, Servicebio, China, GB11020; alpha-smooth muscle actin (α-SMA), 1500, Servicebio, China GB13044) overnight at 4 °C. After three washes with PBS, tissue sections were incubated with secondary antibodies diluted 1:200 for 50 min at room temperature (Cy3-conjugated goat anti-rabbit IgG (H+L) (Servicebio, China; GB21303) and fluorescein isothiocyanate-labeled goat anti-mouse IgG (H+L) (Servicebio, China; GB22301)). After an incubation with DAPI (Servicebio, China; GB1012) and anti-fluorescence quenching seal tablet-diluted buffer (Servicebio, China; GB1401), images of stained tissue sections were captured using a fluorescence microscope (Nikon Eclipse Ti-SR, Japan).

Cells cultured in 96-well plates were washed with PBS (Gibco™, USA). Then, they were fixed and permeabilized with 4% paraformaldehyde (Servicebio, China; HJ194101) and Triton X 100 (Solarbio, China; 9002-93-1). Cells were incubated with a COL1A1 (E8F4L) XP® rabbit mAb (Cell Signaling Technology, USA; 72026S) and anti-periostin antibody (Abcam, USA; ab79946) overnight at 4 °C. After three washes with PBS, the cells were incubated with anti-rabbit IgG (H+L) and F(ab')2 fragments (Alexa Fluor® 488 Conjugate) (Cell Signaling Technology, USA; 4412S) at room temperature in the dark for 1 hour. Images were captured using an Olympus IX70 microscope.

### Isolation and cultivation of CAFs from gastric cancer samples

CAFs were isolated and cultured from gastric cancer samples obtained from patients using primary culture 17. CAFs were identified by the presence of alpha collagen type I (COL1A1). All cells were cultured in DMEM F-12 (Gibco, USA) supplemented with 10% fetal bovine serum (Gibco, USA).

### Western Blotting

Periostin expression was assessed by performing western blot analysis. The primary antibodies we used were as follows: anti-periostin (1:1000; Abcam, ab79946) and anti-GAPDH (1:1000; Cell Signaling Technology, 2118S). The secondary antibody we used was goat anti-rabbit IgG (H+L)-HRP (1:3000; Neobioscience, China, ANR02-2).

### Differentiation of THP-1 cells into M2 macrophages

THP-1 cells were treated with 100 ng/ml PMA (Sigma, 16561-29-8) for 24 h and then rested for 24 h before exposure to 30 ng/ml IL-4 (KEXIN, China; kx20-4) for 24 h. The primers used to identify the M2 phenotype were as follows: *ALOX15* (F: CAGATGTCCATCACTTGGCAG; R: CTCCTCCCTGAACTTCTTCAG); *TGM2* (F: GCAGTGACTTTGACGTCTTTGCCC; R: GTAGCTGTTGATAACTGGCTCC-ACG); and *CD206* (*MRCI;* F: CGAGGAAGAG-GTTCGGTTCACC; R: GCAATCCCGGTTCTCATGGC).

### Transwell migration assay

The Transwell migration assay was performed as previously described 18. The Transwell chamber (aperture 8 μm; Costar, Kennebunk, ME, USA) was used to assess migration. In the absence of fetal bovine serum, 1×10^5^ M2 macrophages were grown in the upper chamber. Next, 2×10^5^ MSCs or CAFs were used as migration attractants and were seeded in the lower chamber. Cell culture medium was discarded after 12 hours. Cells in the upper chamber were washed with PBS, fixed with 4% paraformaldehyde (Servicebio, China; HJ194101) for 10 min, and stained with crystal violet (Solarbio, China; G1063). Finally, the migrating cells were counted and photographed.

### Lentiviral particle preparation and transduction

An NC-expressing cassette was constructed in LV5(EF-1a/GFP&Puro) (GenePharma, Suzhou, China). The inserted sequence was NM_006475.3. The lentivirus was added to the cell culture medium according to the manufacturer's instructions. Culture medium containing puromycin (2 μg/ml) was used to select cells 24 h after transduction. The transduction efficiency was evaluated by detecting GFP expression under a fluorescence microscope (Olympus IX70). Transfection efficiency was then verified using western blotting.

### Immunohistochemical Staining

Sequential pathological sections of GC were incubated with E-cadherin (Servicebio, GB11082), cluster of differentiation (CD)-8 (Servicebio, GB11068-1), PD1 (CST, 86163T), CD163 (Servicebio, GB14027), αSMA (Servicebio, GB111364), CD34 (Servicebio, GB121693), periostin (Abcam, ab79946), and collagen type I alpha 1 chain (COL1A1) (CST, 72066T) antibodies overnight at 4 °C, followed by an incubation with a goat anti-rabbit secondary antibody (Servicebio, G1213) or a goat anti-mouse secondary antibody (Servicebio, G1214) for 1 h at room temperature. Sections were incubated with DAB and imaged under a microscope (Nikon Eclipseci).

### Multistaining registration and positive density analysis

We aligned the images of multistained histological sections and quantified CAFs using the image registration technique 19. The WSIs were first registered onto one slide, which was usually the centroid slide of serial sections, to align the tissue in images and obtain the spatial information for multiple biomarkers in images of immunohistochemical staining. A typical registration process was constructed using Python. Images were downsampled to a size of approximately 500 × 500 pixels and then registered to downsampled target images. The deformation fields were generated and upsampled to the original target image size. Finally, the digital scanned image was aligned with the upsampled deformation field, and the serial sections labeled with antibodies against different biomarkers were spatially aligned to study the distribution of multiple cells. An expression heatmap was generated for each biomarker to calculate colocalization. The annotated regions on the scanned image were dissected into smaller regions of 1000 × 1000 pixels. The marker-positive cell densities were calculated for each small region, and the expression hotspots were easily identified on the heatmap.

### Statistical Methods

The proportions of different cellular subgroups between the tumor samples and AM samples were compared using the two-tailed Mann-Whitney test and unpaired t test with GraphPad Prism 8.4.0 software. Normality tests were performed prior to the t test. For data without equal SD, we used the Mann-Whitney test to compare the ranks. Bars and error bars represent the means and standard errors of the means, respectively.

## Results

### Single-cell gene expression profiling of GC primary tumors and matching adjacent mucosal tissues revealed seven major cell types in the TME

We comprehensively profiled the cell populations in human gastric cancer by generating single-cell gene expression profiles for GC primary tumors and matched adjacent mucosal gastric tissues resected from 8 patients with untreated GC. The carcinoma types of eight patients included three intestinal-type GCs, three diffuse-type GCs, and two mixed-type GCs (Table S1).

**Figure 1:**
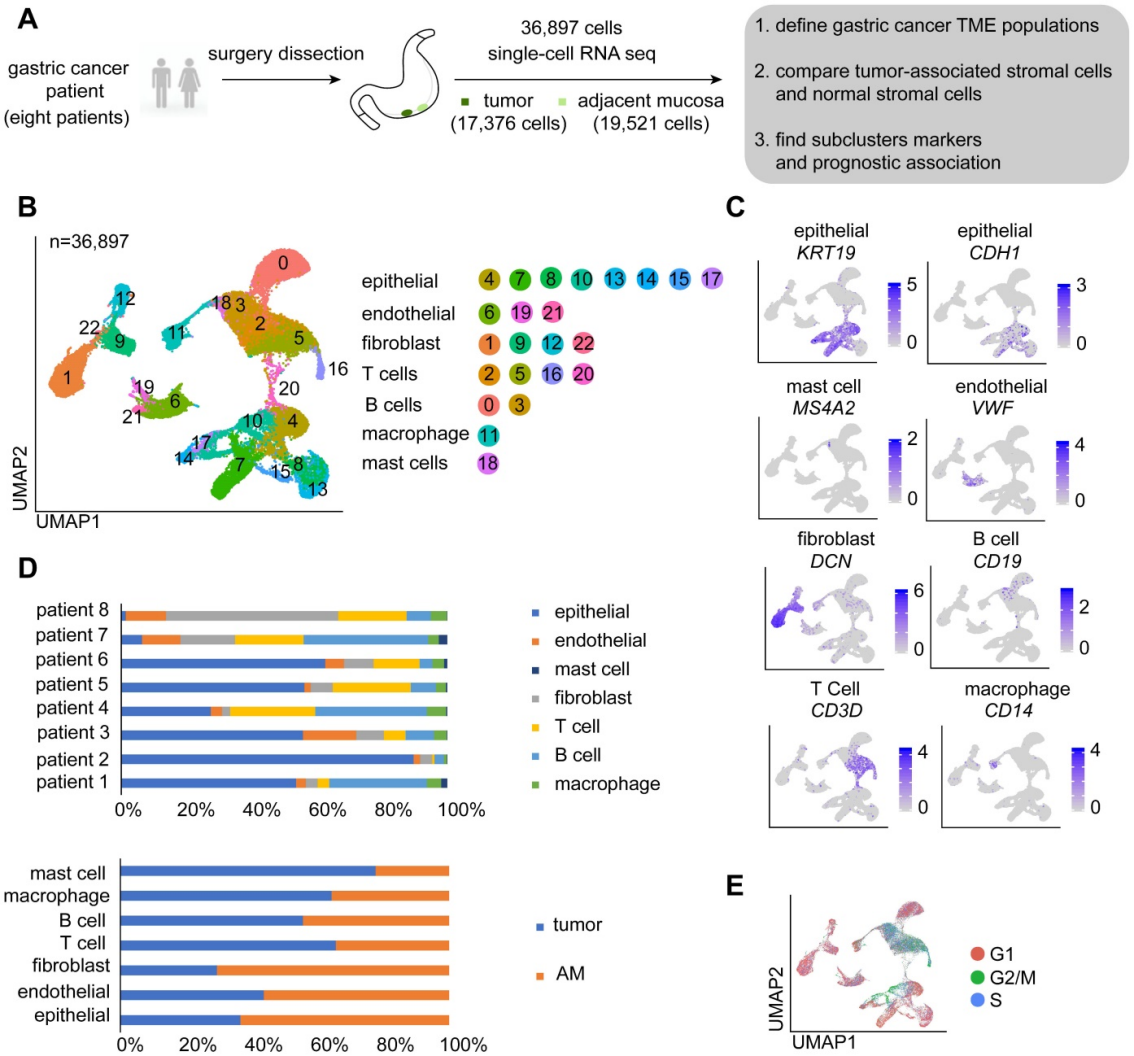
** Overview of the single cells isolated from eight primary gastric cancer (GC) lesions and matching adjacent mucosal (AM) samples. A.** Summary of the workflow used to collect the specimens and perform single-cell transcriptome sequencing in the tumor microenvironment (TME) of GC. **B.** Uniform Manifold Approximation and Projection (UMAP) plot of the analyzed single cells. Each color represents one cluster. Annotated cell types are listed below. **C.** UMAP plot color-coded (gray to blue) to represent the expression levels of the marker genes for the seven cell types, which are listed beyond the UMAP plot. **D.** The distribution of cells derived from different patients or different sample origins. **E.** UMAP clustering of the 36,897 cells, with each color representing a different stage of the cell cycle.

A total of 36,897 cells were acquired, of which 17,376 cells were obtained from GC and 19,521 were obtained from AM tissues (Figure 1A; Table S2). We performed dimensional reduction and unsupervised clustering of cells to identify cell groups based on their expression patterns 20. As shown in Figure 1B, 23 cell clusters were identified. The annotation of these clusters resulted in the separation of seven cell types 9, 21-23: epithelial cells, endothelial cells, fibroblasts, T cells, B cells, macrophages, and mast cells (Figure 1B-C). Remarkably, cells identified as epithelial cells, endothelial cells, fibroblasts, T cells, and B cells belong to multiple clusters, indicating that these cell types may be heterogeneous (Figure 1B).

We showed that the cells derived from both tumor and normal tissues from different patients were split into seven categories (Figure 1D). As proliferation is one of the main characteristics of tumor cells, we performed a cell cycle analysis using a cell cycle gene scoring method. The majority of T cells and a small fraction of epithelial cells derived from GC samples were in G2, M, or S phase of the cell cycle 24, whereas other cells were in G1 phase of the cell cycle (Figure 1E).

### Tumor-derived epithelial cells are heterogeneous among patients

**Figure 2:**
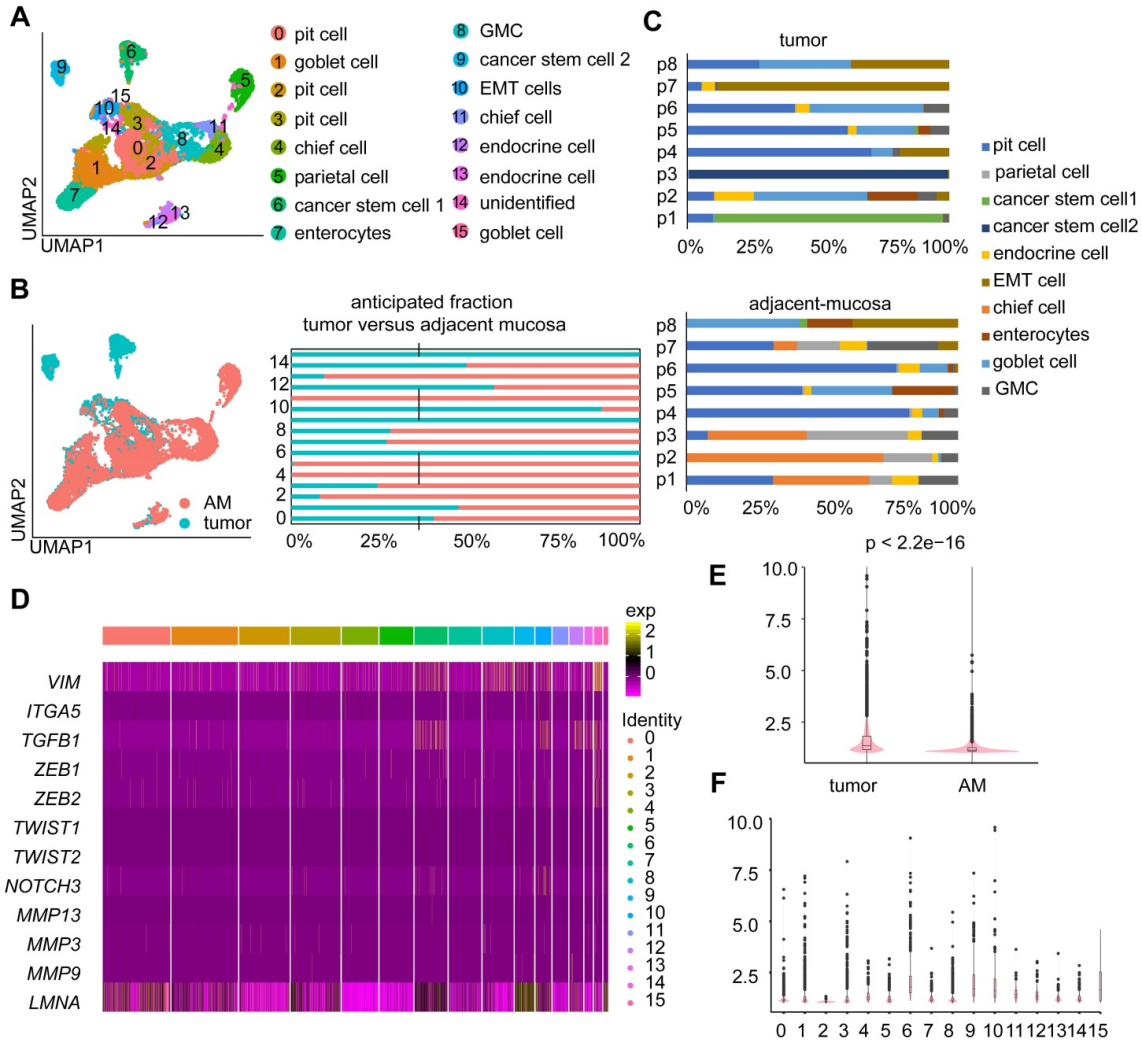
** Heterogeneity in the gene expression of GC-associated epithelial cells. A.** UMAP visualization of 11,187 epithelial cells. Each color represents one cluster (see cluster ID panel). “endocrine cell” represents “enteroendocrine cell”. **B.** UMAP plot and bar plot of epithelial cells. Colors represent sample origins, either tumor-derived (Clusters 0, 1, 6, 9, 10, 12, 14, and 15) or AM-derived samples. **C.** The distribution of different epithelial cell subgroups derived from the tumor samples or AM samples from different patients. P1-P8 represent patient 1-patient 8. Colors represent the epithelial cell subgroups.** D.** Heatmap showing the expression levels of various partial epithelial-to-mesenchymal transition (p-EMT) genes in different clusters. **E.** p-EMT signature scores for tumor and normal cells. **F.** p-EMT signature scores across all clusters.

Epithelial cells were the most abundant cell type in both the tumor and AM samples (Table S2; Figure S1A). We investigated the cellular heterogeneity of epithelial cells by performing an unsupervised clustering analysis of 11,187 epithelial cells. We identified 16 clusters (Figure 2A), of which eight clusters were mostly derived from tumor samples (Figure 2B). Based on the expression of marker genes (Figure S1B), we identified nine epithelial cell subtypes: enterocytes, pit cells, chief cells, parietal cells, enteroendocrine cells, goblet cells, antral basal gland mucous (GMC) cells, and cancer stem cells 1 and 2 (Figure 2A). These cell subtypes were identified in both tumor and adjacent samples, except for cancer stem cell 1” and cancer stem cell 2”. These two tumor cell subgroups were mainly derived from tumor samples from patient 1 and patient 3, indicating that the cancer epithelial cells were highly heterogeneous among patients (Figure 2C).

Next, we conducted a differential gene expression analysis and GO enrichment analysis to explore how expression states differed between tumor-derived epithelial cells and AM-derived epithelial cells. Genes that were upregulated in tumor-derived epithelial cells were enriched in malignant biological properties, including cell proliferation, response to wounding, and positive regulation of NF-kappa B transcription factor activity (Figure S1C).

Moreover, a fraction of epithelial cells derived from tumor samples exhibited increased expression levels of partial epithelial-to-mesenchymal transition (p-EMT) signature genes (Figure 2D-E). Interestingly, tumor-derived Cluster 10 showed the highest p-EMT expression levels (Figure 2F), indicating that it represented a subgroup of cells undergoing active p-EMT. Therefore, we refer to cells present in Cluster 10 as “EMT cells”.

### A fraction of GC-associated endothelial cells exhibit endothelial-to-mesenchymal transition (EndMT) signatures

**Figure 3:**
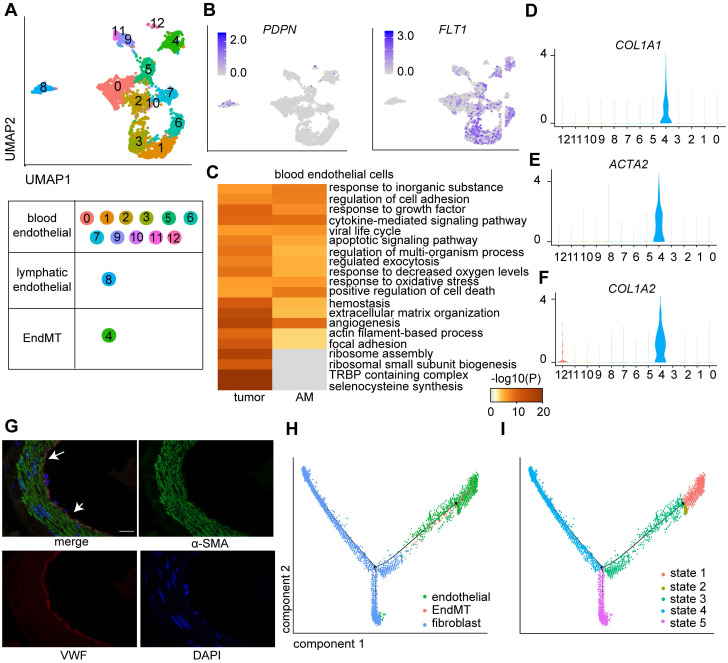
**Clusters of endothelial cells in eight pairs of gastric tumor and adjacent normal samples. A.** UMAP plot color-coded for 13 clusters of endothelial cells. 'EndMT' refers to EndMT cells. **B.** UMAP plot color-coded (gray to blue) to represent the expression levels of the marker genes: lymphatic endothelial cells, podoplanin (*PDPN*); blood endothelial cells, fms-related receptor tyrosine kinase 1 (*FLT1*); tumor samples (n = 8); AM samples (n = 8).** C.** Enriched Gene Ontology (GO) terms for the genes that were differentially expressed between the tumor-derived and AM-derived blood endothelial cells. P values are shown (from gray to red). **D-F.** Violin plot showing the distribution of collagen type I alpha 1 chain (*COL1A1*)*,* collagen type I alpha 2 chain (*COL1A2*) and actin alpha 2 (*ACTA2*) among the different clusters. **G.** Images of immunofluorescence staining of representative GC tumors with antibodies against von Willebrand factor (VWF) (red) and alpha-smooth muscle actin (α-SMA) (green). Scale bar, 100 μm. **H-I.** Pseudotime analysis of endothelial cells and fibroblast cells derived from tumor samples inferred by Monocle2. Each point corresponds to one single cell. Each color represents one cell subgroup (**H**). Each color represents one cell state (**I**).

Endothelial cells are tightly regulated by the TME, which is essential for tumor growth and metastasis 25. We explored the heterogeneity of endothelial cells by further clustering the 2,973 endothelial cells into 13 clusters (Figure 3A; Table S2), among which eight clusters were derived from tumor samples, and the remaining clusters were derived from AM samples (Figure S2A). Cluster 8 was identified as a lymphatic endothelial cell line. The remaining clusters were identified as blood endothelial cells (Figure 3A-B).

We performed a differential gene expression analysis of cells of tumor origin and compared gene expression with cells of AM origin to dissect the roles of lymphatic endothelial cells and blood endothelial cells in GC progression. We identified 243 genes (p < 0.01) that were differentially expressed between tumors and AM-derived lymphatic endothelial cells. For example, *SPRY1* was significantly upregulated in tumor-derived lymphatic endothelial cells (Figure S2B).

Regarding blood endothelial cells, tumor-derived blood endothelial cells exhibited upregulated expression of genes that were significantly associated with the GO terms “regulation of cell adhesion” and “extracellular matrix organization” (Figure 3C). AM-derived blood endothelial cells displayed upregulated expression of genes that were associated with “myeloid leukocyte activation”, “blood vessel development”, “regulation of cell adhesion” and “regulation of cytokine production” (Figure S2C), indicating that AM-derived blood endothelial cells also exhibited increased angiogenesis and cytokine production 26.

Interestingly, cells in Cluster 4 expressed both an endothelial marker gene (*VWF*) and fibroblast marker genes (*COL1A1,* collagen type I alpha 2 chain (*COL1A2*), and actin alpha 2 (*ACTA2*)) at high levels (Figure 3D-F). We also identified a group of cells coexpressing VWF and α-SMA in tumor samples by performing immunofluorescence staining of samples from a different cohort of patients with gastric cancer (Figure 3G). We performed a pseudotime analysis of these two cell groups to investigate the transition between endothelial cells and fibroblasts. Cells in Cluster 4 were located in an intermediate stage on the pseudotime trajectory between endothelial cells and two groups of fibroblasts (Figure 3H-I).

The endothelial-to-mesenchymal transition (EndMT) is a critical step in vasculogenesis during embryonic development. Several studies have suggested that the EndMT also plays a role in tumor progression 27. We performed a differential gene expression analysis of cells in tumors compared with those in adjacent mucosal samples in this cluster to explore the role of cells in Cluster 4 (named the EndMT cluster) in GC tumorigenesis. We showed that upregulated genes in tumor-derived EndMT cells were associated with the functional terms “TNF-alpha/NF-kappa B signaling complex 6” and “collagen fibril organization” (Figure S2D), suggesting that EndMT cells are involved in sprouting angiogenesis in GC 28.

### eCAFs promote invasion and are associated with shorter overall survival of TCGA cohort

Cancer-associated fibroblasts (CAFs) have been recognized as an important component of the tumor microenvironment because of their diverse roles in promoting tumor progression.

**Figure 4:**
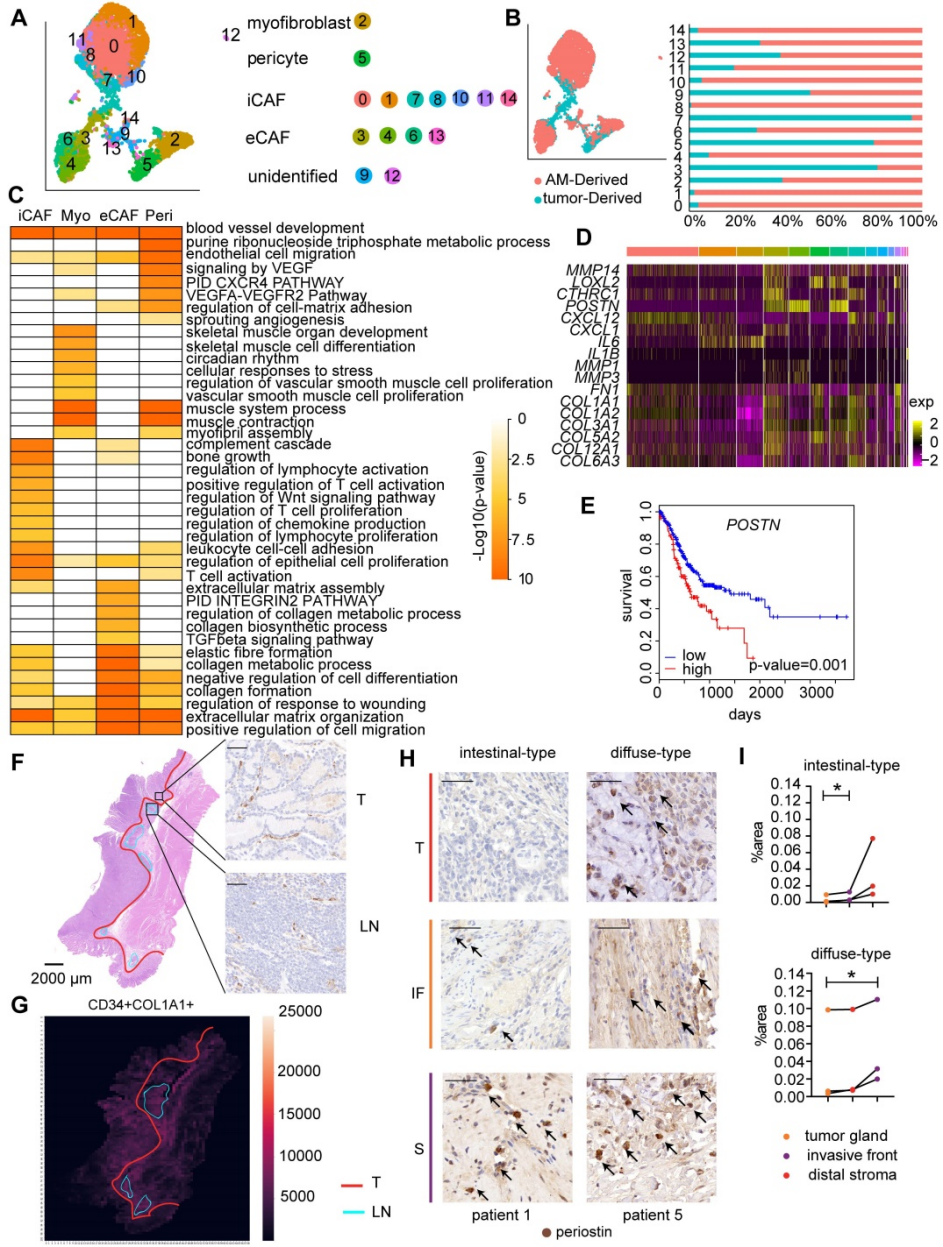
** Fibroblast clusters in the GC and AM samples. A.** UMAP plot showing 14 clusters of fibroblast cells colored according to different clusters identified here. **B.** UMAP plot of the tumor-derived and AM-derived fibroblast cells: tumor samples (n = 8) and AM samples (n = 8). Bar chart showing the composition by sample origin as the total percentage of each cell type per sample. The X-axis represents the cell proportion, and the Y-axis represents clusters. **C.** Heatmap showing the selected significant terms for the genes differentially expressed in the four subgroups of tumor-derived fibroblasts. Colors represent the P values (from white to orange). 'Myo' represents myofibroblasts. 'Peri' represents pericytes.** D.** Heatmap of the expression levels of the genes associated with invasion, cell migration, and extracellular matrix remodeling in different clusters of fibroblast cells.** E.** Overall survival curves of the patients with stomach cancer in The Cancer Genome Atlas (TCGA), stratified by the mRNA expression levels of the *POSTN* gene. The red line shows the survival curve of the patients exhibiting high *POSTN* expression levels in the tumor samples (for the top 30% of all samples); the blue line shows the survival curve of the remaining patients (P value = 0.001). **F.** Hematoxylin and eosin (H&E) staining and immunohistochemical staining for cluster of differentiation (CD)-34 (marker of iCAFs) in tumor tissues from patient 6 (scale bar, 50 μm). Upper right panel: CD34-positive staining in the tumor gland (T). Lower right panel: CD34-positive staining in the lymphoid nodule-like structure (LN). Arrows indicate the positive staining for CD34. **G.** Heatmap showing the density of positive staining for CD34 and COL1A1 in the tumor tissues from patient 6. The area inside the yellow line represents the tumor gland (T). The area inside the blue line indicates the lymphoid nodule-like structure (LN). The remaining area is the stroma (S). **H.** Tumor sections from patients with intestinal-type and diffuse-type GC showing how periostin-positive cells are distributed in the tumor gland (T), invasive front (IF) and distal stroma (S). Cells were stained for periostin in tumor tissues from patient 1 and patient 5. Arrows indicate the positive staining. **I.** Proportions of the periostin-positive area in the tumor gland (T), invasive front (IF) and distal stroma (S). Upper panel: Proportion in each patient with intestinal-type GC. The P value was calculated based on the paired t test (P = 0.0377). Lower panel: Proportion in each patient with diffuse-type GC. The P value was calculated based on the paired t test (P = 0.0464).

We identified four main fibroblast subgroups in both GC samples and AM samples based on the expression of specific cellular markers: myofibroblasts, pericytes, extracellular matrix CAFs (eCAFs), and immunomodulatory CAFs (iCAFs) (Figure 4A-B; Figure S3A) 11, 29. We also examined the marker gene expression levels in these CAF subgroups using TCGA bulk RNA-seq data. The expression levels of gene markers for the same cell type were highly correlated with each other (Figure S4). Additionally, the resident fibroblast subsets in AM samples expressed the same marker genes as the corresponding CAF subsets, but did not express genes involved in pathways for CAF activation, such as proliferation, angiogenesis, inflammation, and extracellular matrix (ECM) remodeling (Figure S5A; Table S3). Based on these results, the CAF subsets educated by the surrounding tumor cells gain enhanced protumor abilities compared to distal AM-derived fibroblasts.

We performed an analysis of differentially expressed genes (DEGs) in all four fibroblast subpopulations to understand the distinct roles of the four fibroblast subgroups. GO terms enriched for genes that were expressed at high levels in myofibroblasts included muscle contraction, vascular smooth muscle cell proliferation, and blood vessel development (Figure 4C), confirming that they played a regulatory role in the endothelial differentiation process as myofibroblasts. The GO analysis of genes that were upregulated in pericytes revealed enrichment of the term “blood vessel development”, consistent with the definition that pericytes are cells that enwrap capillaries and promote their survival (Figure 4C, Table S3). Interestingly, consistent with the activated sprouting angiogenesis observed in tumors 25, we showed that the fraction of pericytes was significantly increased in tumor samples (P=0.0117) (Figure S3B; Table S4-S5).

iCAFs expressed *CXCL12, IL6*, and *CXCL14* at high levels (Table S4), which are marker genes of inflammatory CAFs (iCAFs) described in other solid tumors. In addition, genes that were upregulated in tumor-derived iCAFs were associated with the regulation of the inflammatory response, namely, *IL6ST, NFKBIA, NT5E, SERPINF1,* and* NFKBIZ* (Figure 4C; Table S3), suggesting that they play roles in communicating with immune cells.

Importantly, we identified a previously unreported CAF subpopulation characterized by high expression of *POSTN* (Figure S3A), a gene encoding a protein that supports the adhesion and migration of epithelial cells, in addition to promoting cancer stem cell maintenance and metastasis 30. We showed that this CAF subset expressed genes involved in ECM remodeling at high levels, including genes encoding collagens and collagen metabolic enzymes (Figure 4C, Figure S3C); therefore, we defined them as extracellular matrix CAFs (eCAFs). Tumor-derived eCAFs (Cluster 3) are characterized by high expression of genes associated with tumor invasion (*MMP14, LOXL2,* and *POSTN*) (Figure 4D), indicating that eCAFs constitute an essential component of the TME for tumor metastasis. We analyzed the correlation between gene expression profiles and prognosis in the bulk transcriptome of 423 TCGA GC samples to evaluate whether the presence of high levels of eCAFs is associated with a worse prognosis of gastric cancer. As shown in Figure 4E, patients with high *POSTN* gene expression levels experienced shorter overall survival, even after stratifying for the stromal fraction in the tumor samples (Cox proportional hazards model, p=0.001). Together, these results suggest that eCAFs represent an essential CAF subpopulation that promotes metastasis and subsequently affects the prognosis of patients with GC.

We performed immunohistochemical staining for CAF marker genes in serial pathological sections and combined the results of markers of interest using image registration analysis to investigate the distribution of the four CAF subsets in GC. We divided the tumor tissue into three parts: tumor gland (T), distal stroma (S), and lymphoid-like structures (L) (Figure S6A). Myofibroblasts and pericytes (stained with αSMA and COL1A1 antibodies, respectively) were evenly distributed throughout the tumor tissue, including the tumor gland and distal stroma area (Figure S6A). iCAFs were enriched in the tumor gland and were located around the tumor cells (Figure 4F). iCAFs were identified in lymphoid nodule-like structures (Figure 4F), indicating that they may be involved in regulating lymphocytes. Notably, iCAFs were more enriched in the tumor gland area than in the distal stroma area (Figure 4G). The periostin-positive area was increased in the order of tumor gland, invasive front, and distal stroma (Figure 4H-I, Figure S6B), indicating that eCAFs mainly reside in the distal stroma area. The different spatial distributions of eCAFs suggested their distinct roles in the tumor microenvironment.

### The TME of GC presents an immunosuppressive state

**Figure 5:**
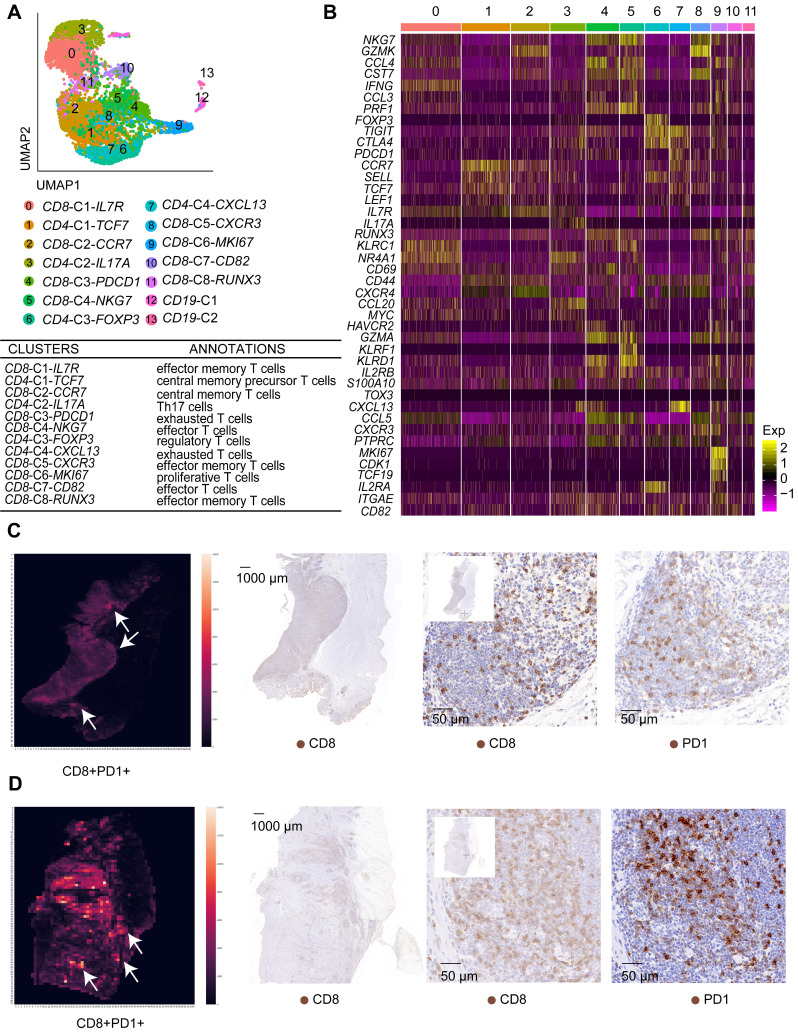
** T cell clusters in tumor samples and AM samples. A.** UMAP plot of T cells showing 14 clusters annotated in different colors. **B.** Heatmap of marker gene expression in each annotated cell subgroup.** C.** Heatmap showing the density of positive staining for CD8 and PD1 and IHC staining for CD8 and PD1 in the tumor tissue from patient 6 (intestinal-type). **D.** Heatmap showing the density of positive staining for CD8 and PD1 and IHC staining for CD8 and PD1 in the tumor tissue from patient 5 (diffuse-type).

T cells are the most highly enriched immune cells in the TME. We investigated the functional states of T cells in the GC TME by analyzing 6,874 T cells in tumor and AM tissues (Table S2). We identified 14 clusters using an unsupervised clustering analysis (Figure 5A). T cells from the tumor samples clustered separately from T cells in AM samples in the UMAP plot (Figure S7A), indicating that their functional properties differ significantly.

Based on gene expression signatures, we identified 12 main groups, including eight clusters of CD8+ T cells and four clusters of CD4+ T cells (Figure 5A-B; Figure S7B). We further annotated these T cell clusters into eight T cell subtypes by examining the expression of marker genes. The eight subtypes are CD8+ effector memory T cells, CD8+ effector T cells, CD4+ central memory precursor T cells, CD8+ central memory T cells, Th17 cells, regulatory T cells, exhausted T cells, and proliferative T cells (Figure 5A, Table S6).

Importantly, we identified two exhausted T cell subgroups, *CD8*-C3-*PDCD1* and *CD4*-C4-*CXCL13*. The *CD8*-C3-*PDCD1* subgroup exhibited the substantial upregulation of the *PDCD1* and *HAVCR2* genes, while the *CD4*-C4-*CXCL13* subgroup presented the substantial upregulation of the* PDCD1, TIGIT,* and* CTLA4* genes (Figure 5B). Notably, the *CD8-C3-PDCD1* subgroup expressed *CD69* and various cytotoxic genes, such as *GZMH, GZML*, and *NKG7*, at high levels, indicating that it may be activated (Figure 5B). We confirmed whether exhausted T cells could be identified in GC tissues by performing immunohistochemistry for CD8 and PD1 in serial pathological sections and the image registration analysis. T cells expressed both CD8 and PD1 in the lymphoid structures of GC (Figure 5C-D).

CD4+ central memory precursor T cells, *CD4*-*C1-TCF7*, were identified as the most abundant T cell subgroup in tumor samples but not in AM samples (Figure S8A-B). In the AM samples, effector T cells and effector memory T cells were identified as the most abundant T cell subgroups (Figure S8A-B). In addition, a greater number of Tregs infiltrated tumor samples than AM samples (Figure S8I; Table S5). Together, these findings clearly indicate that the immediate immune function of T cells is impaired in the GC TME.

We clustered 8,090 B cells, of which 4,493 cells were derived from tumor samples, to investigate the functional roles of tumor-infiltrating B cells in GC (Table S2). Thirteen clusters were identified. Clusters 2, 6, and 7, which were characterized by the expression of the* FCER2* and *CR2* genes (Figure S9A-B), were annotated as naïve B cells. Cluster 12 expressed *BCL6* (Figure S9A-B), suggesting that it comprises the germinal center B cell subset. The remaining clusters expressed immunoglobulin (Figure S9A-B), suggesting that they constituted plasma B cells.

We showed that the gene expression profiles differed between tumor-derived B cells and AM-derived B cells (Figure S9C), indicating that B cells were reprogrammed by the TME. Next, we analyzed differential gene expression between tumor-derived naïve B cells and AM-derived naïve B cells. The genes expressed by tumor-derived B cells exhibited a stronger association with “cellular responses to stress”, “positive regulation of cell death” and “intrinsic apoptotic signaling pathway”, suggesting that naïve B cells in the tumor samples underwent apoptosis (Figure S9D).

Macrophage density is associated with a poor prognosis for patients with many solid tumors 31. We explored the role of macrophages in the TME by performing an unsupervised clustering analysis of our scRNA-seq data for 1,481 macrophages, of which 952 cells were derived from tumors (Table S2). Similarly, tumor-derived macrophages were distributed differently from AM-derived macrophages (Figure S9E). We obtained 11 clusters, of which Clusters 2 and 6 were annotated as tumor-associated macrophages and clusters and 1 and 5 were annotated as myeloid-derived suppressor cells (Figure S9F-G). Genes that were upregulated in tumor-derived macrophages were enriched in functions of leukocyte migration, myeloid leukocyte activation, and antigen processing and presentation (Figure S9H), suggesting that tumor-derived macrophages play roles in regulating immune cell infiltration in the TME 32.

### Dynamic communication among different TME components

We conducted cell-cell communication analysis based on our single-cell data using the CellPhoneDB toolkit to investigate the communication between different fibroblast subgroups and other components in the tumor microenvironment 33. CellPhoneDB infers cell-cell communication networks based on curated ligand-receptor interactions and the expression of ligands and receptors in different cell types from scRNA-seq data. We identified strong interactions among endothelial cells, fibroblasts, and macrophages in both tumor and AM-derived samples (Figure S10A-B). CAF-endothelial communication was mediated by angiogenesis-associated *VEGFA*-receptor pairs (Figure S10C), confirming that CAFs play a critical role in the activation of angiogenesis. Moreover, the epithelial cells in the tumor exhibited a stronger interaction with other TME components than the epithelial cells in the AM (Figure S10A-B), indicating that the tumor cells were actively regulated by other components in the TME. For example, tumor epithelial cells expressing *PDGFA* interacted with *PDGFRA*-expressing fibroblasts (Figure S10D).

**Figure 6:**
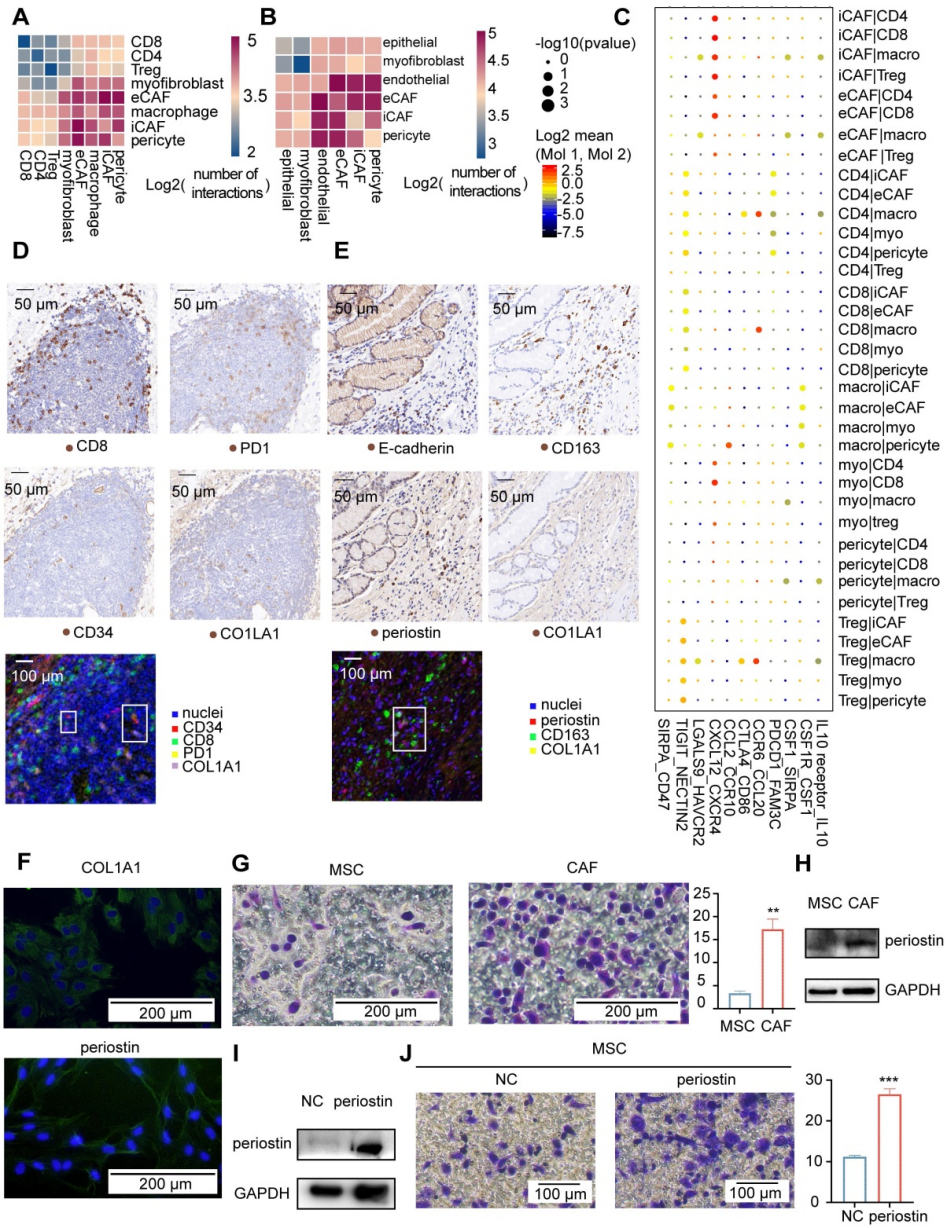
** Communication among tumor microenvironment components. A-B.** Heatmap showing interactions between fibroblast subgroups and immune cells (A) or other stromal cells (B). The color represents the number of interactions (blue to red); n=8 tumors.** C.** Selected specific interactions between CAF subgroups and immune cells in tumors. The size indicates the p values, and the color indicates the mean values of the receptor/ligand pairs between two clusters. **D.** IHC staining and pseudofluorescence synthesized by multistaining registration for CD8, PD1, CD34, and COL1A1 in the tumor tissue from patient 6. The box indicates adjacent cells. **E.** Images of IHC staining and pseudofluorescence synthesized by multistaining registration for E-cadherin, CD163, periostin, and COL1A1 in the tumor tissue from patient 6. The box indicates adjacent cells. **F.** Immunofluorescence staining for COL1A1 and periostin in the fifth generation of CAFs. **G.** Crystal violet staining of M2 macrophages in response to MSCs and CAFs. Bar plot showing the average crystal violet-stained area in Transwells calculated using ImageJ software. The p value was calculated using the unpaired t test (P value = 0.0038). **H.** Western blot results showing a comparison of the periostin and GAPDH bands between MSCs and CAFs. **I.** Western blot results showing a comparison of the periostin and GAPDH bands between NC-MSCs and periostin-overexpressing MSCs. **J.** Crystal violet staining of M2 macrophages that were recruited in response to NC-MSCs and periostin-overexpressing MSCs. Bar plots showing the average crystal violet-stained area in the Transwells calculated using ImageJ software. The p value was calculated using the unpaired t test (P value = 0.0004).

The recruitment of immune cells to the TME is a process that can be targeted during cancer therapy. We analyzed the cell-cell communications between different subgroups of stromal and immune cells in the TME to provide insights into immune cell recruitment in the TME (Figure 6A-B). Our analysis showed that iCAFs communicated with *CD4*+ T cells, *CD8*+ T cells, Tregs, and macrophages through receptor-ligand interactions associated with chemokines, inflammatory cytokine responses, and immune modulation (Figure 6C). Taken together, iCAFs play a role in the recruitment of lymphocytes. We tested this hypothesis by analyzing the abundance of immune cell types in bulk RNA sequencing data from TCGA gastric cohort. As shown in Figure S10E, the expression levels of gene markers for iCAFs were associated with the abundance of lymphocytes. We also identified that the main subpopulation of CAFs residing in lymphoid nodule-like structures was iCAFs (Figure 6D), which were located around CD8-positive and PD1-positive T cells, indicating that iCAFs are involved in regulating T cells.

For comparison, *POSTN* expression was more strongly correlated with the macrophage fraction in TCGA tumor samples (Figure S10E), which is the most highly correlated gene even after correction for the overall stromal fraction in tumor samples. In addition, *POSTN* expression was more strongly correlated with protumorigenic M2 macrophages than antitumorigenic M1 macrophages in tumor samples (Figure S10E). We compared the sites of periostin and CD163 (an M2 macrophage marker) expression using immunohistochemical methods to investigate the spatial relationship between eCAFs and M2 macrophages. Periostin-positive cells correlated spatially with CD163-positive cells (Figure 6E). We further confirmed that eCAFs functioned in recruiting M2 macrophages by performing functional experiments in cultured cells. First, we obtained M2 macrophages *in vitro* by inducing Thp1 cells to differentiate into M2 macrophages (Figure S10F). Next, we obtained eCAFs from gastric cancer samples. We first isolated and cultured all CAFs from gastric cancer samples. All second-generation gastric cancer sample-derived cells expressed COL1A1 (Figure S10G), and approximately 18% of cells expressed periostin (Figure S10G-H), indicating that we obtained gastric cancer CAFs and that some CAFs were eCAFs. Interestingly, after passaging these cells four times, almost all CAFs expressed periostin (Figure 6F). eCAFs may have a higher proliferation potential than other CAFs in cell culture.

We used fifth-generation CAFs as eCAFs to perform the Transwell migration assay, with mesenchymal stem cells (MSCs) serving as the negative control to identify whether eCAFs recruited M2 macrophages. We showed an increased number of migrated M2 macrophages in response to eCAFs compared with MSCs (Figure 6G). Periostin was expressed at significantly higher in eCAFs than in MSCs (Figure 6H). As the *POSTN* gene was correlated with M2 macrophages in TCGA analysis, we further investigated the role of periostin in recruiting M2 macrophages by overexpressing periostin in MSCs (Figure 6I). Periostin-overexpressing MSCs increased the migration of M2 macrophages (Figure 6J). Based on these results, eCAFs exhibited enhanced chemotaxis of attracting M2 macrophages *in vitro*. Taken together, iCAFs and eCAFs have different functions in immune cell recruitment within the tumor microenvironment.

In addition, communication between macrophages and lymphocytes was also identified (Figure S10A). The recruited macrophages expressed *CTLA4, LGALS9*, and *HAVCR2*, all of which are likely involved in suppressing the function of lymphocytes expressing *CD86* and *HAVCR2* (Figure 6C).

### TME components that infiltrated diffuse-type GC construct a microenvironment favoring cancer progression

**Figure 7:**
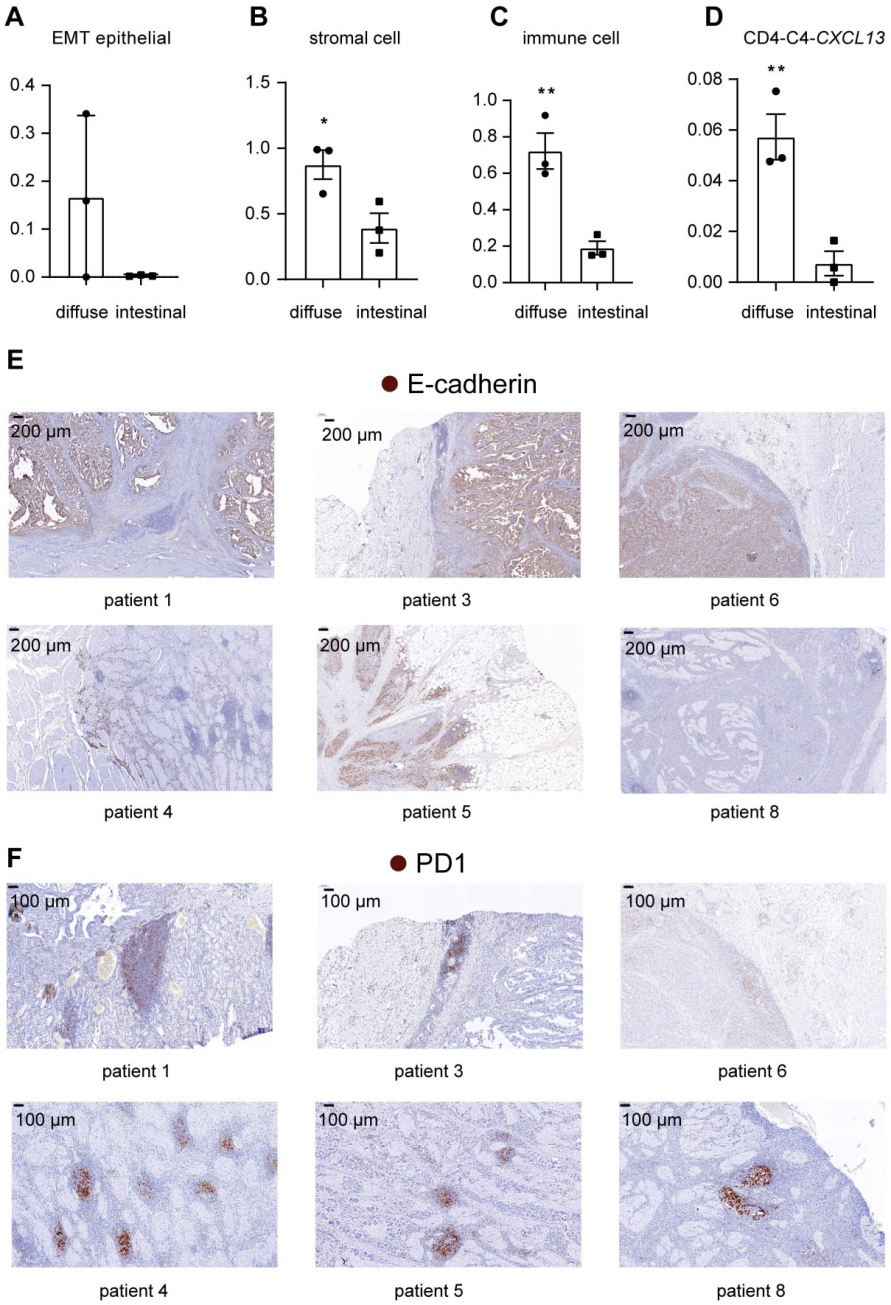
** Comparison of cell subgroup fractions between two histopathological types of gastric cancer. A.** Fraction of EMT epithelial cells relative to total nonimmune cells in 6 tumor samples. **B.** Fraction of all stromal cells relative to the total cells in 6 tumor samples. “*” represents a p value<0.05; unpaired Student's t test. **C.** Fraction of immune cells relative to total cells in 6 tumor samples. “**” represents a p value<0.01; unpaired Student's t test. **D.** Fraction of *CD4-C4*-*CXCL13* cells relative to the total immune cells in 6 tumor samples. “**” represents a p value<0.01; unpaired Student's t test. **E.** IHC staining for E-cadherin in tumor tissues from patients with intestinal-type GC (patients 1, 3 and 6) and diffuse-type GC (patients 4, 5 and 8). **F.** IHC staining for PD1 in tumor tissues from patients with intestinal-type GC (patients 1, 3 and 6) and diffuse-type GC (patients 4, 5 and 8).

We compared cell subgroup ratios between patients with diffuse and intestinal GC to investigate the correlation of cellular heterogeneity with pathological classifications in GC. The fractions of EMT epithelial cells were higher in two of the three patients with diffuse-type GC than in the three patients with intestinal-type GC (Figure 7A), confirming the presence of more undifferentiated cells in diffuse-type GC. As shown in Figure 7B, the diffuse-type tumor samples also exhibited a higher fraction of stromal components (endothelial, CAF, and immune cells, p=0.038).

In addition, the fraction of immune cells was higher in diffuse-type tumor samples than in intestinal-type tumor samples (Figure 7C) (p=0.0073), suggesting that diffuse-type tumor samples had a higher degree of immune cell infiltration. Within subtypes of immune cells, we showed that the relative abundance of *CXCL13*-expressing T cells was higher in diffuse-type tumors (Figure 7D) (p=0.0081), suggesting that diffuse GC had a more suppressive immune microenvironment. We also compared the pathological features of intestinal-type and diffuse-type tumors. Clear boundaries were observed between the tumor and stromal regions in intestinal-type tumors but not in diffuse-type tumors. E-cadherin was expressed at lower levels in diffuse-type tumors than in intestinal-type tumors, indicating that the fraction of EMT epithelial cells was higher in diffuse-type tumors (Figure 7E). Lymphoid nodule-like structures developed along the margins of the tumor region in intestinal-type tumors (Figure 7F). We showed that lymphoid nodule-like structures of diffuse-type tumors resided near tumor cells, and the number of lymphoid structures in diffuse-type tumors was higher than that in intestinal tumors (Figure 7F). PD1 was only expressed in the lymphoid structures (Figure 7F), indicating that more exhausted T cells infiltrated in diffuse-type tumors. Taken together, diffuse-type GC is characterized by a 'Four High' phenotype (high EMT, high stromal cell, high immune cell infiltration, and high T cell exhaustion), indicating that more metastatic tumor cells and the immunosuppressive TME may account for the poor prognosis of patients with diffuse GC.

## Discussion

Our study revealed the heterogeneity of the GC TME, revealing the significant variability in abundance and expression signatures among tumor epithelial cells and other TME cell subsets. We mainly focused on the diversity of CAFs, which regulate different aspects of the biology of the TME. iCAFs chemoattract and regulate the function of T cells by secreting IL6 and CXCL12, which are similar to the inflammatory CAFs identified in other solid tumors 34, 35. For example, CAF-S1 found in breast cancer recruits CD4+CD25+ T cells by secreting CXCL12 36. CAF-S1 is also correlated with macrophage infiltration. In contrast to CAF-S1 in breast cancer, in our study, eCAFs, rather than iCAFs, were significantly correlated with tumor-associated M2-like macrophages. We also observed a spatial association between eCAFs and M2-like macrophages. eCAFs, which express *POSTN*, remodeled the extracellular matrix in the TME, suggesting their roles in promoting invasion and metastasis. eCAFs were also significantly correlated with a poor prognosis, suggesting that the increased infiltration of eCAFs forms a metastasis-friendly niche by degrading the ECM and chemoattracting tumor-associated M2-like macrophages. Similar to previous studies 10, 37, we did not determine the exact M1 and M2 macrophage subsets. We observed that a TAM cluster (Cluster 2) expressed *CD163* (Table S4), indicating that these cells are characterized as M2-like macrophages. Further studies on eCAFs and TAMs in GC are needed to understand how they function in metastasis and how they can be used as potential therapeutic targets to inhibit metastasis. We annotated Cluster 5 of fibroblasts as pericytes based on the expression of the* RGS5* gene. The origin of pericytes remains controversial. Recent studies have shown that activated fibroblasts transdifferentiate into pericytes in the TME 38. However, the origin of these pericytes identified in GC requires further exploration.

According to recent studies, senescent fibroblasts develop a senescence-associated secretory phenotype, including inflammatory factors, chemokines, and extracellular matrix elements, and promote tumor growth 39. Interestingly, iCAFs in GC tumors upregulated the expression of the* CDKN1A* gene (encoding P21 protein) and expressed inflammatory factors, chemokines, and ECM elements in our study.

The abundance and expression signature of tumor epithelial cells presented significant interpatient variability, such as cancer stem cell 2, which was derived only from patient 3. Variations in tumor epithelial cells are driven by various mutations among patients 35. Unlike tumor epithelial cells, the diversity of CAFs only presented a difference in abundance across patients. Diverse CAF subsets were significantly correlated with the biological behaviors of other stromal cells in the GC TME. Combining classical therapy with treatments targeting CAFs may reduce the supportive effect of the TME on tumor cells and produce a favorable outcome in the majority of patients.

Disease recurrence after curative resection of advanced GC results in a poor prognosis 2. The reason for the recurrence and metastasis of advanced GC remains unclear. We observed that the resident fibroblast counterparts of four CAF subsets were identified in the adjacent mucosal samples. These fibroblast subsets were less activated than the corresponding CAF subsets. We hypothesize that the wound repair process and the constant inflammation caused by curative resection activate resident fibroblasts to form a tumor-supportive milieu and may correlate with the local recurrence of GC. Combining postoperative chemotherapies with the inhibition of CAF activation may reduce recurrence and improve the prognosis of patients with advanced GC.

Lauren subtypes are associated with the prognosis. Compared to patients with intestinal-type GC, patients with diffuse-type GC typically have a younger onset age and lower 5-year survival rates. Diffuse-type tumors are poorly differentiated, exhibit a greater infiltration depth, and have a higher degree of lymphatic vessel invasion 40. Our findings presented here provide clear evidence for greater infiltration of metastatic tumor cells and stromal cells in diffuse-type tumors, which may explain why diffuse-type tumors are more aggressive.

TCGA molecular classification, which affects prognostic predictions and personalized therapy, has become a popular method for determining the pathological subtypes of GC in patients 41. Determining how the TME is constituted in each TCGA subtype may provide more insights into the diagnosis and treatment of patients with GC. We aligned our findings by classifying our samples according to TCGA molecular classification. As only one patient was classified into the EBV subtype, we did not observe significant differences among the four molecular subtypes. Further studies should include a larger patient cohort to understand how the TME is constituted in each TCGA subtype.

Similar to other scRNA-seq studies of solid tumors 42, 43, we also identified that p-EMT epithelial cells and proliferating T cells were enriched in GC tumors, highlighting the universal importance of these two cell subsets across different types of cancer. We also showed that a small number of p-EMT epithelial cells and proliferating T cells were identified in AM samples. We hypothesized that the reason why a few AM-derived epithelial cells exhibited a higher p-EMT score may be that those cells remain unhealed, as the gastric mucosa of patients with GC may be in an inflammatory state 44. Moreover, these AM-derived proliferative T cells might be intraepithelial lymphocytes residing in the gastric mucosa 45.

Unlike previous scRNA-seq studies of GC 9, 10, 37, which focused on the heterogeneity of malignant cells or immune cells among different subtypes of GC, our study mainly focused on elucidating the classification and function of different CAF subsets in GC. Moreover, we validated the spatial distribution of CAF subsets in GC using multistaining registration analysis and confirmed the chemotaxis ability of eCAFs in attracting M2 macrophages *in vitro*. The limitation of our analysis is the small number of patient samples included in our study, resulting in poor yields of several cell subsets. Our findings require verification in a larger cohort of patients. The digestion process may also affect the transcriptome of cells. Furthermore, we studied CAF heterogeneity based on scRNA-seq data collected from dissociated tumor samples. Although we validated the spatial distribution of CAF subsets in GC using multistaining registration analysis and confirmed the chemotaxis ability of eCAFs in attracting M2 macrophages *in vitro*, the isolation and enrichment of other CAFs was challenging for us due to sample availability. For example, for iCAF isolation, negative selection of CD31 must be performed to exclude CD31-expressing endothelial cells, which requires a tissue size that is not feasible for early-stage gastric cancers in patients undergoing surgery. By culturing all COL1A1-expressing CAFs, we also only obtained eCAFs, as they may have a higher proliferation potential. Therefore, methods to isolate and enrich iCAFs and other CAFs from gastric cancer samples for functional studies require further exploration.

## Conclusions

We profiled approximately 37,000 cells from tumors and adjacent mucosal tissues from eight patients with GC using single-cell RNA sequencing. We mainly discussed CAFs in the GC TME, including the classification, function, origin, interaction with other cell subsets, and spatial distribution in different pathological types. These results reveal the unique roles of CAFs in regulating different aspects of the biology of the TME, including immune modulation, invasion, migration, and angiogenesis. Importantly, we showed that eCAFs presented an enhanced chemotaxis ability of attracting M2 macrophages and were associated with a poor prognosis of patients with GC, indicating that inhibiting eCAF activation may be a potential therapeutic target for patients with GC.

## Supplementary Material

Supplementary figures.

Supplementary table 1.

Supplementary table 2.

Supplementary table 3.

Supplementary table 4.

Supplementary table 5.

Supplementary table 6.

## Abbreviations

GC: Gastric cancer
TME: Tumor microenvironment
scRNA-seq: Single-cell RNA sequencing
EndMT: Endothelial-to-mesenchymal transition
CAF: Cancer associated fibroblast
ECM: Extracellular matrix
AM: Adjacent mucosa
iCAF: inflammatory cancer-associated fibroblast
eCAF: excellular matrix CAF
UMAP: Uniform approximation and projection
DEGs: Differentially expressed genes
GO: gene ontology
IHC: Immunohistochemistry
interleukin (IL)-6: IL6
C-X-C motif chemokine ligand 12: CXCL12
periostin: POSTN
transcripts per million: TPM
von Willebrand factor: VWF
alpha-smooth muscle actin: α-SMA
cluster of differentiation (CD)-8: CD8
collagen type I alpha 1 chain: COL1A1
antral basal gland mucous: GMC
partial epithelial to mesenchymal transition: p-EMT
collagen type I alpha 2 chain: COL1A2
actin alpha 2: ACTA2
Hematoxylin and eosin: H&E
The Cancer Genome Atlas: TCGA
