# Supplementary material for: Single-cell RNA sequencing reveals a pro-invasive cancer-associated fibroblast subgroup associated with poor clinical outcomes in patients with gastric cancer
Source: Theranostics. 2022 Jan 1;12(2):620–38. doi: 10.7150/thno.60540 (PMC8692898; doi:10.7150/thno.60540)
Supplement: Supplementary file 1 — Supplementary figures. [file thnov12p0620s1.pdf]

Supplemental legends and figures

Single-cell RNA sequencing reveals a pro-invasive cancer-associated fibroblast subgroup associated with poor clinical outcomes in patients with gastric cancer

Xuechun Li1#, Zhao Sun2#, Gongxin Peng3#, Yi Xiao4, Junchao Guo4, Bin Wu4, Xiaoyi Li4, Weixun Zhou5, Jiarui Li2, Zhe Li6, Chunmei Bai2, Lin Zhao2*, Qin Han1*, Robert Chunhua Zhao1,7*, Xiaoyue Wang3*

1 Institute of Basic Medical Sciences Chinese Academy of Medical Sciences, School of Basic Medicine Peking Union Medical College, Peking Union Medical College Hospital, Center of Excellence in Tissue Engineering Chinese Academy of Medical Sciences, Beijing Key Laboratory (No. BZO381), Beijing, People's Republic of China.

2 Department of Medical Oncology, Peking Union Medical College Hospital, Chinese Academy of Medical Sciences, Beijing, China.

3 Center for Bioinformatics, Institute of Basic Medical Sciences, Chinese Academy of Medical Sciences, School of Basic Medicine, Peking Union Medical College, Beijing, 100005, China.

4 Department of General Surgery, Peking Union Medical College Hospital, Chinese Academy of Medical Sciences, Beijing, China

5 Department of Pathology, Peking Union Medical College Hospital, Chinese Academy of Medical Sciences, Beijing, China

6 Department of Gynecologic Oncology, National Cancer Center/ National Clinical Research Center for Cancer/Cancer Hospital, Chinese Academy of Medical Sciences and Peking Union Medical College, Beijing, China

7 School of Life Sciences, Shanghai University, 99 Shangda Road, Shanghai 200444, China

#Xuechun Li, Zhao Sun and Gongxin Peng contributed equally to this work.

*Correspondence to Lin Zhao, Peking Union Medical College Hospital (Dongdan campus), No.1 Shuaifuyuan Wangfujing Dongcheng District, Beijing, China 100730; wz20010727@aliyun.com, Qin Han, Institute of Basic Medical Sciences Chinese Academy of Medical Sciences, 5 Dong Dan San Tiao, Beijing, China 100005; hanqin@ibms.pumc.edu.cn, Robert Chunhua Zhao, Institute of Basic Medical Sciences Chinese Academy of Medical Sciences, 5 Dong Dan San Tiao, Beijing, China 100005; zhaochunhua@ibms.pumc.edu.cn or Xiaoyue Wang, Institute of Basic Medical Sciences Chinese Academy of Medical Sciences, 5 Dong Dan San Tiao, Beijing, China 100005; wxy@ibms.pumc.edu.cn.

Supplemental file 1: Table S1. Patient information.

Supplemental file 2: Table S2. Numbers of seven cell types identified in the eight patients included in this study.

Supplemental file 3: Table S3. Gene Ontology (GO) analysis.

Supplemental file 4: Table S4. Statistics-related information. The first seven sheets provide statistical information on positive marker genes in seven cell types, including p values, average log2-fold changes, and adjusted p values. The eighth sheet contains statistical information for the comparison of every fraction between tumors and adjacent mucosal samples.

Supplemental file 5: Table S5. Numbers of different cell subsets identified in the eight patients included in this study.

Supplemental file 6: Table S6. Marker genes for different T cell subsets.

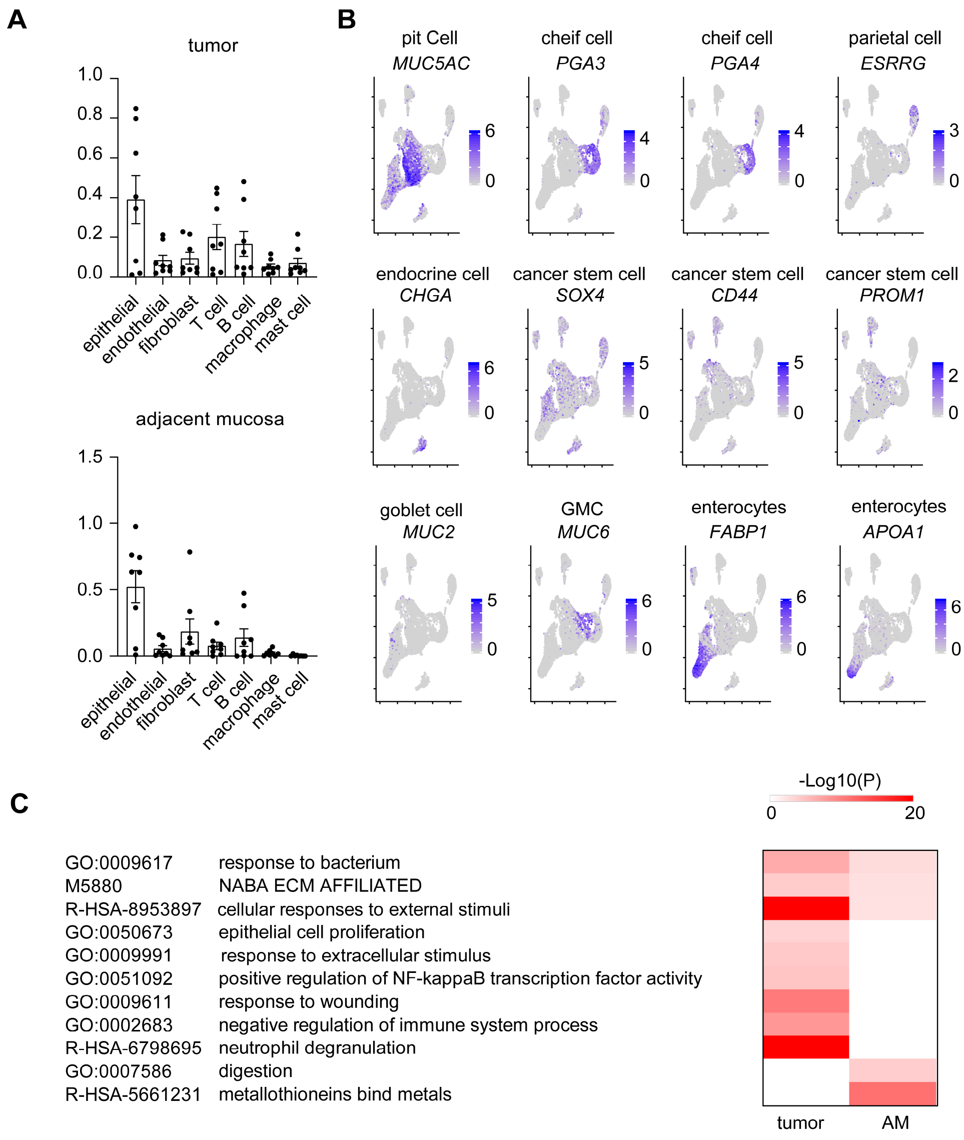

Figure S1. Clustering of epithelial cells. **A.** Fractions of seven different main cell types in eight tumor samples and adjacent mucosal (AM) samples: frequencies relative to all tumor-derived cells and AM-derived cells identified here are shown. n=8 tumors, n=8 AM samples. **B.** UMAP plot color-coded (gray to blue) for marker gene expression. n=8 tumors, n=8 AM samples. **C.** Heatmap showing the selected terms from the top 100 GO terms (based on p value) within tumor-derived epithelial cells and AM-derived epithelial cells. The color distinguishes the p value (from gray

to red).

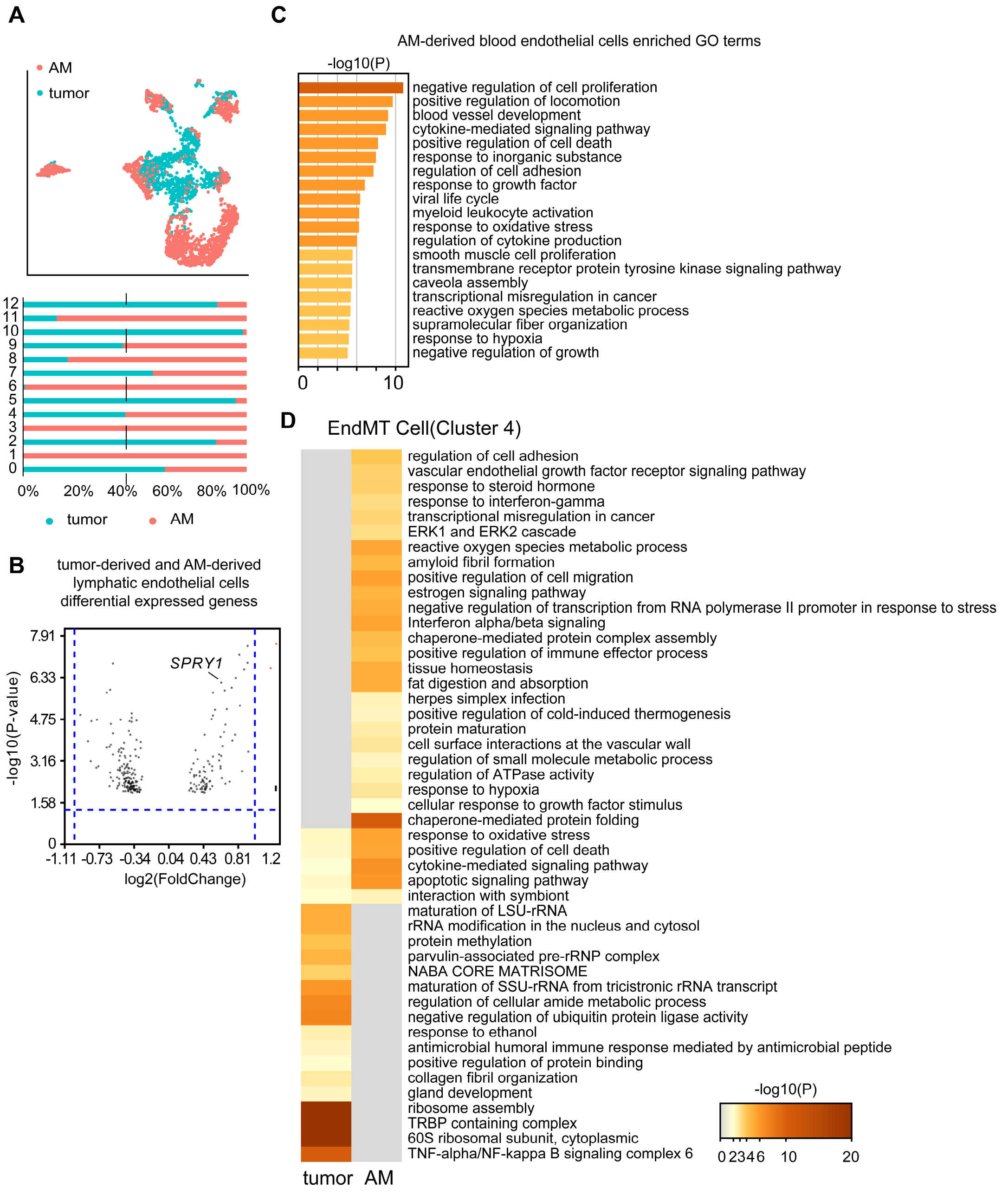

Figure S2. Clustering of endothelial cells. **A.** UMAP plot color-coded for the distribution pattern of tumor-derived (Clusters 0, 2, 4, 5, 7, 9, 10, and 12) and adjacent mucosa (AM)-derived endothelial cells (Clusters 1, 3, 6, 8, and 11). n=8 tumors, n=8 AM samples. Bar chart showing the composition by sample origin as the total percentage of each cell type per sample. The X-axis represents the cell proportion, and

the Y-axis represents clusters. **B.** Volcano plot showing differentially expressed genes between tumor-derived blood endothelial cells and AM-derived cells. The cut p value was 0.05, and the cut log2(fold change) was from -1 to 1. **C.** Bar plot showing the top 10 GO terms enriched in AM-derived blood endothelial cells (based on p value). **D.** Heatmap showing the terms with the best p value within tumor-derived EndMT cells and AM-derived EndMT cells as their representative terms. The cells in the heatmap are colored according to their p values.

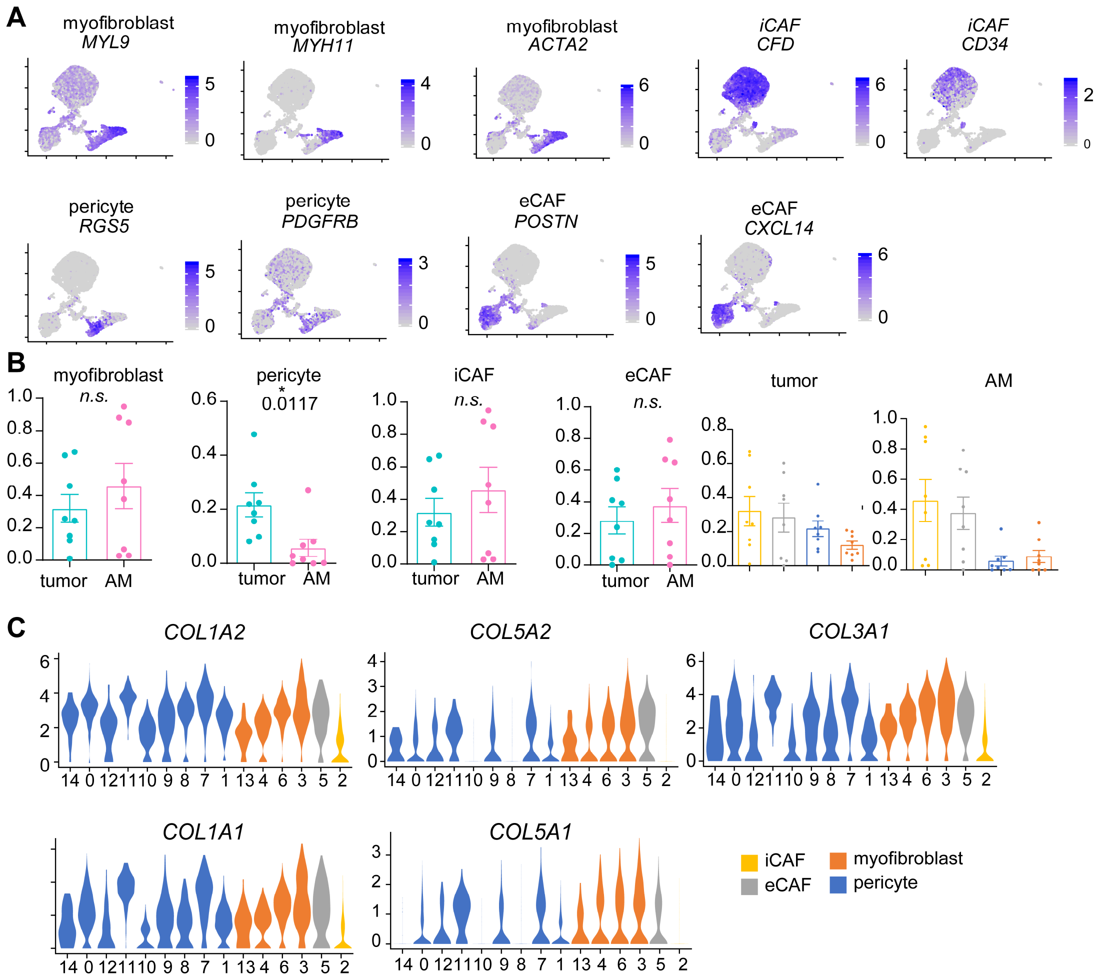

Figure S3. Clustering of fibroblasts **A.** UMAP plot of color-coded fibroblast cells (gray to blue) for marker gene expression. n=8 tumors, n=8 AM samples. **B.** Comparison of four fibroblast subgroups between tumor samples and AM samples ("*": p value<0.05, ns: not significant); relative fractions are calculated relative to all

tumor-derived fibroblasts and AM-derived fibroblasts; n=8 tumors, n=8 AM samples. The p values were calculated using an unpaired t test. Fractions of four fibroblast subgroups in either tumor or AM samples. **C.** Violin plot showing the expression levels of collagen genes in each cluster.

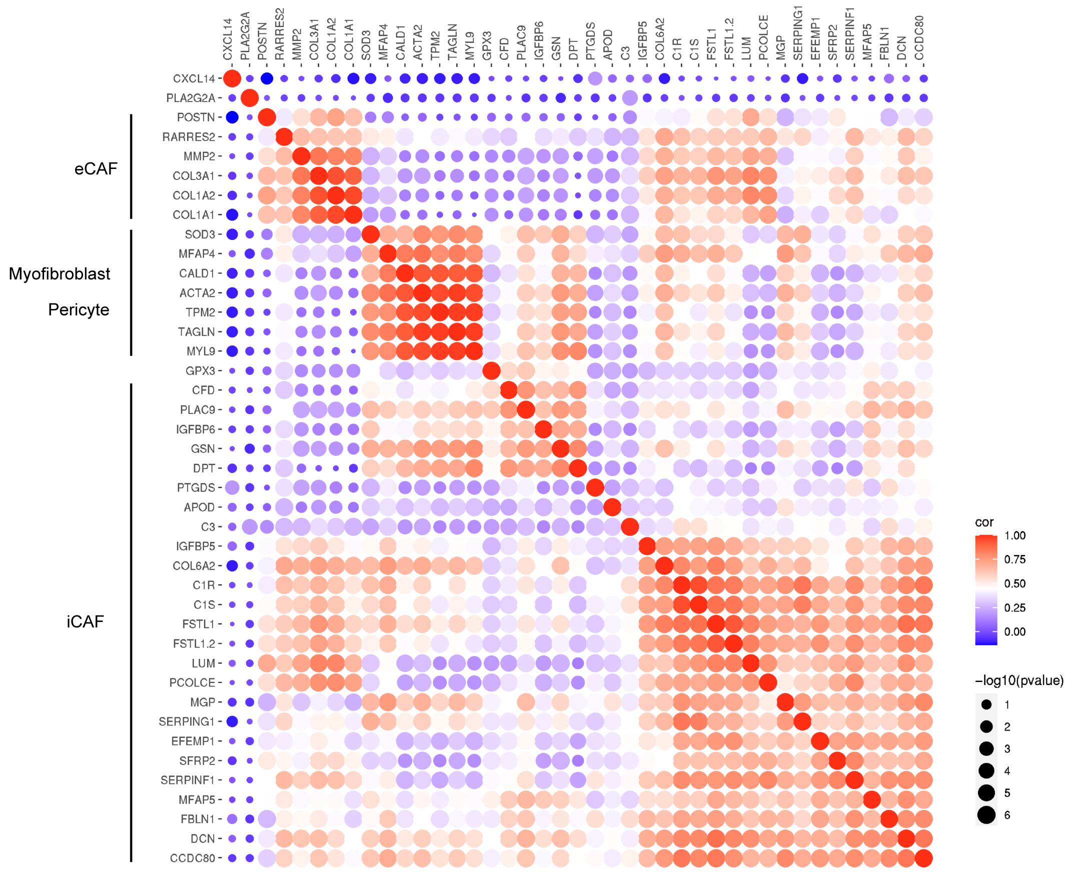

Figure S4. Correlations of the expression of all marker genes for the four CAF subsets in TCGA bulk RNA-seq data calculated using the Pearson correlation method. Colors indicate the coefficients from blue to red. The size of the dots indicates the p values.

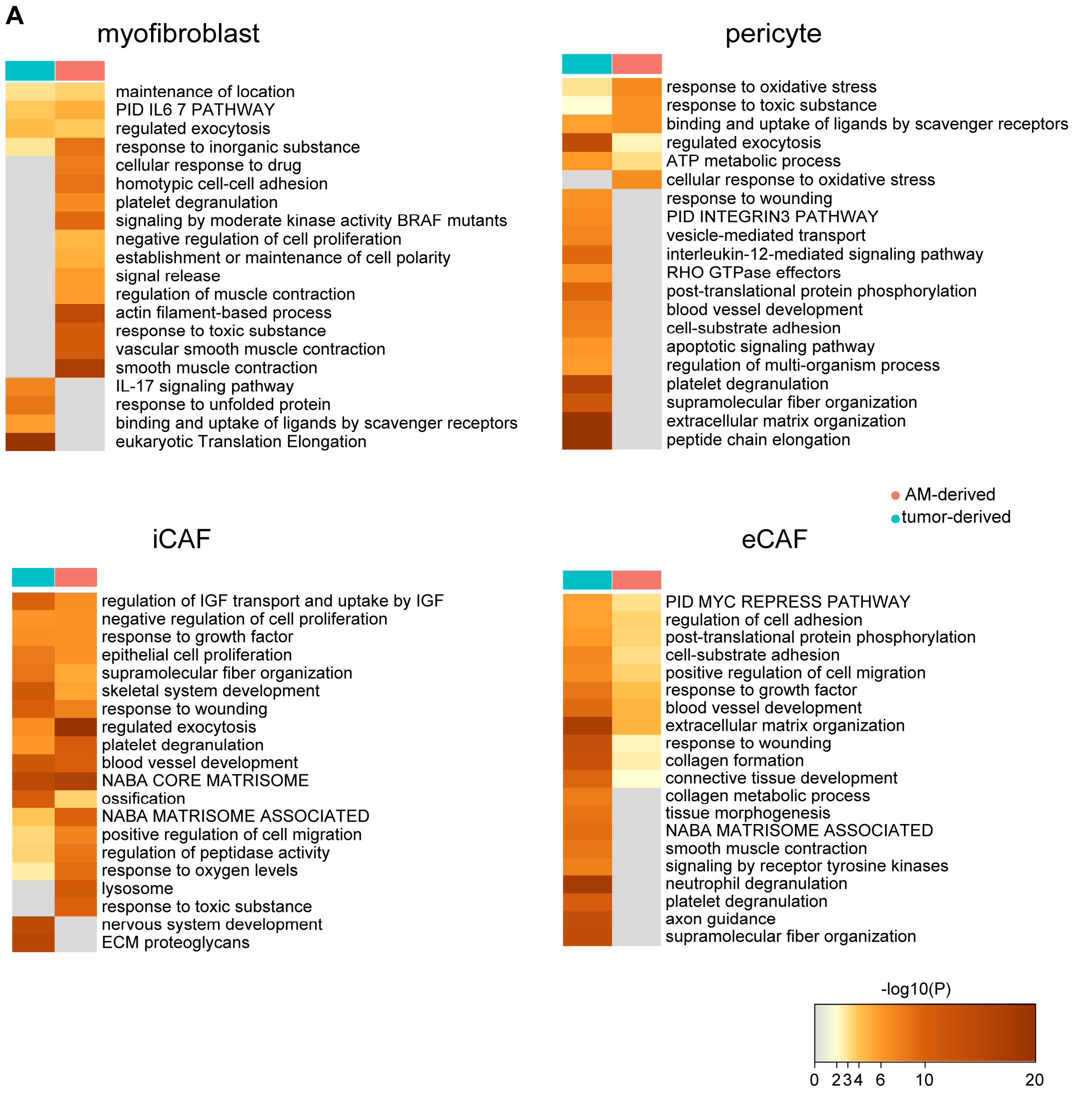

Figure S5. Clustering of fibroblasts **A.** Heatmap showing the terms with the best p value within tumor-derived fibroblasts and AM-derived fibroblasts as their representative terms. The heatmap cells are colored according to their p values.

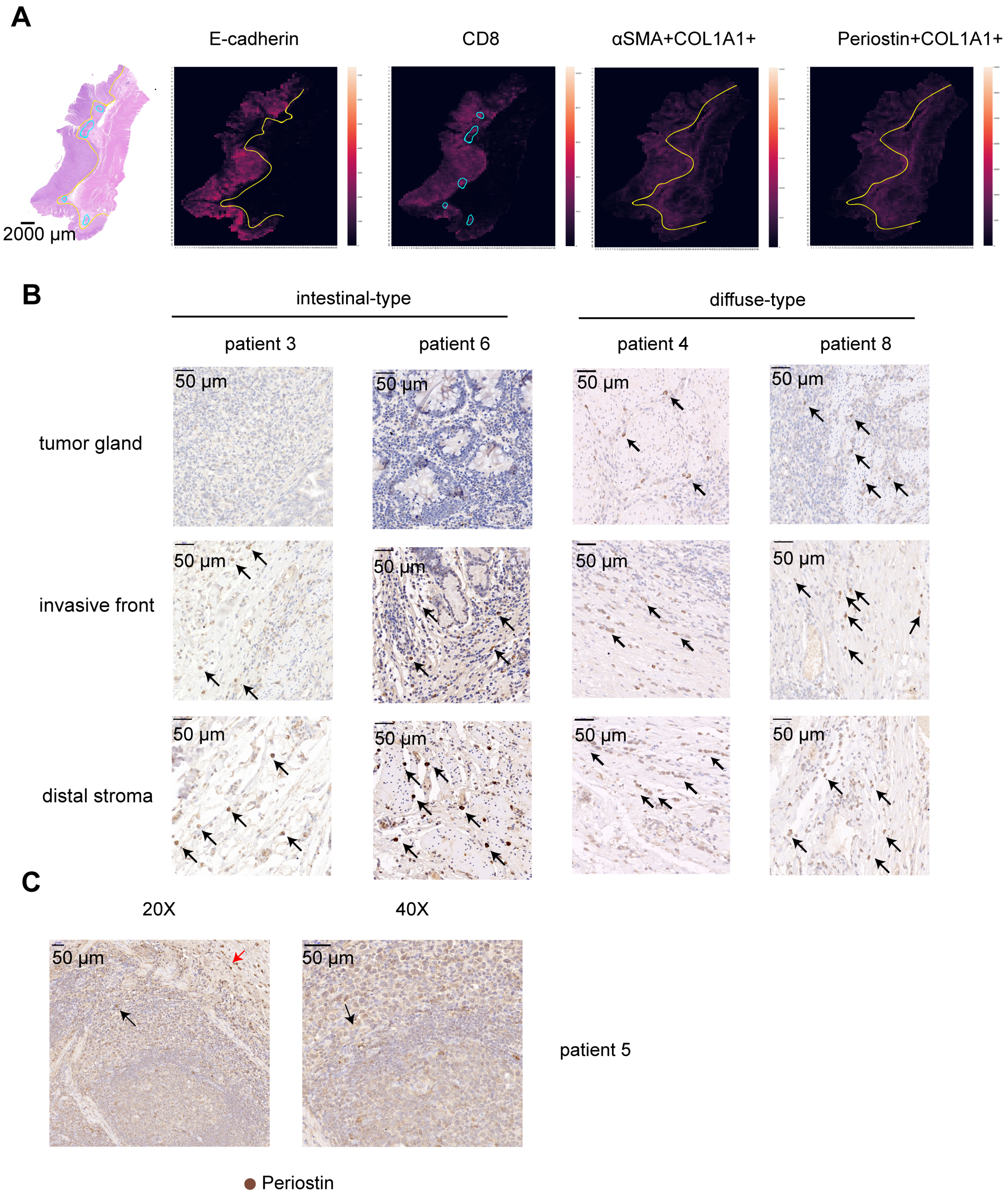

Figure S6. Distribution of the four fibroblast subgroups in GC tumors. **A.** H&E staining of tumor tissues from patient 6 and heatmaps showing the density of positive staining for markers of epithelial cells, T cells, and fibroblast subgroups. The yellow line divides the tumor tissue into two parts: the tumor gland (T) and distal stroma (S). The blue line indicates lymphoid nodule-like structures (LNs). E-cadherin staining of epithelial cells. CD8 staining of CD8 T cells. αSMA+COL1A1+ staining in

myofibroblasts and pericytes. Periostin+COL1A1+ staining in eCAFs. **B.** IHC staining for periostin in tumor tissue from patients with intestinal-type and diffuse-type GC. Arrows indicate positive staining. **C.** IHC staining for periostin in tumor tissues from patient 5. Arrows indicate positive staining of tumor cells.

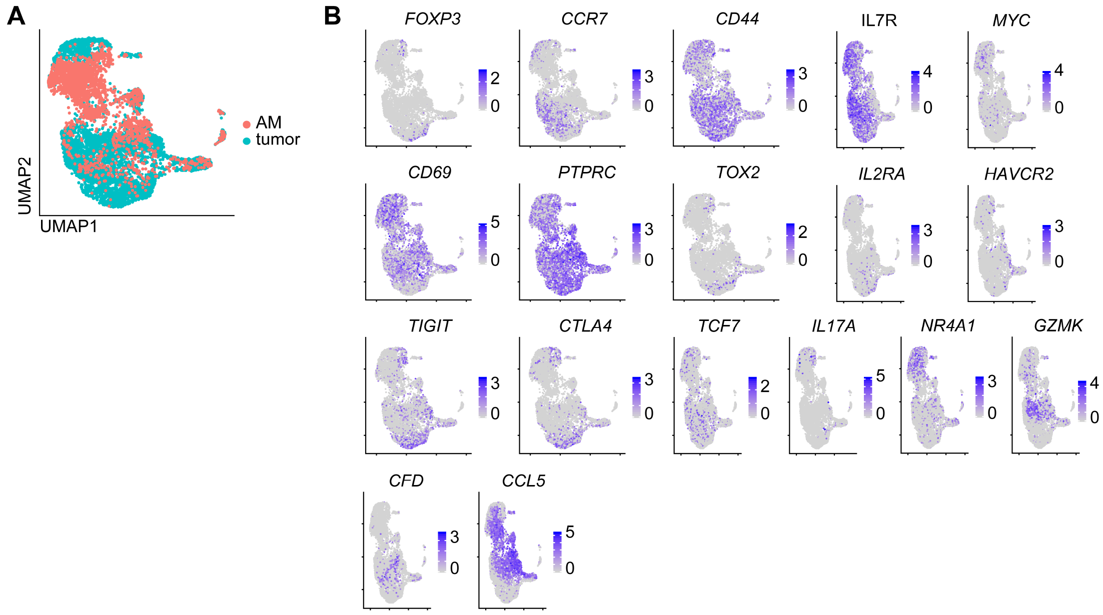

Figure S7. Subclustering of T-cells **A.** UAMP plot color-coded for the distribution pattern of tumor-derived and adjacent mucosa (AM)-derived T cells. n=8 tumors, n=7 AM samples. **B.** UAMP plot color-coded (gray to blue) for marker gene expression in T cells. N=8 tumors, n=7 AM samples.

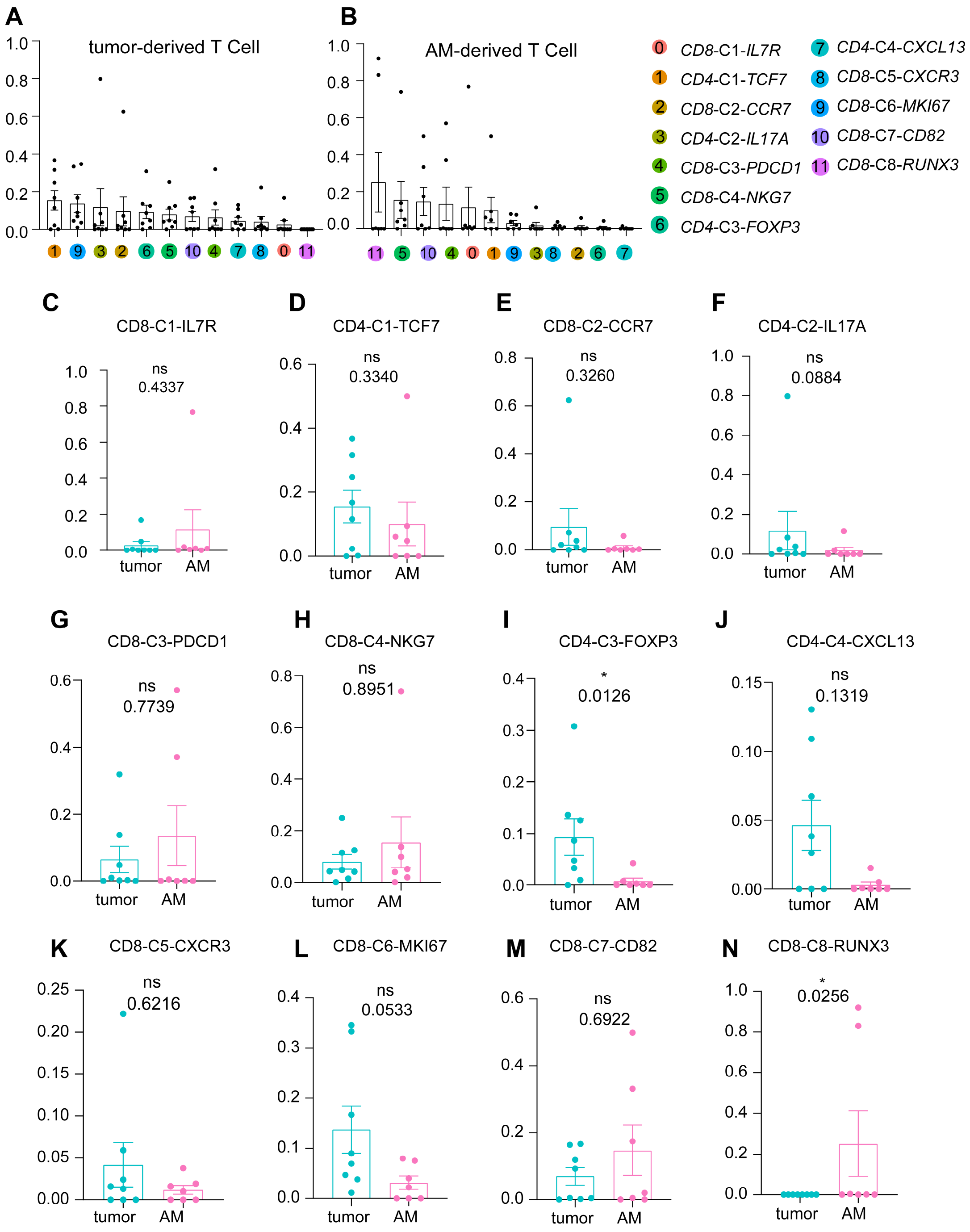

Figure S8. Comparison of the T cell subgroup fractions. Fractions of the T cell subgroups relative to the total T cells in eight tumor samples (**A**) or seven AM samples (**B**). **C-N.** Fraction of each T cell subgroup in the tumor-derived and AM-derived samples; "*" represents p <0.05. n.s. represents not significant. n=8 tumors,

n=7 AM samples. A two-tailed Mann–Whitney U test was performed for all comparisons.

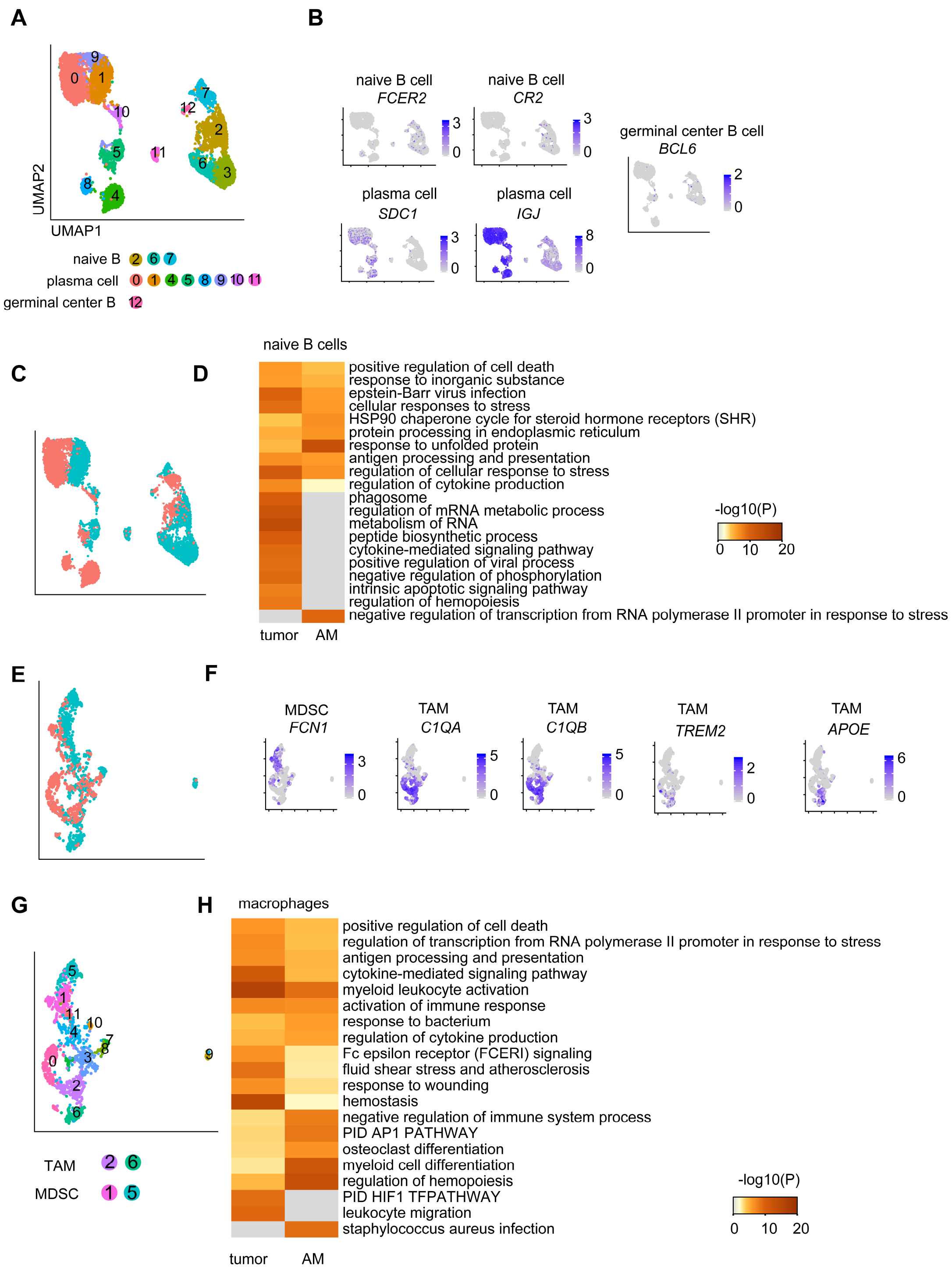

Figure S9. Clustering analysis of B cells and macrophages. **A.** UMAP plot of B cells showing 13 clusters colored according to the clusters. **B.** UMAP plot color-coded

(gray to blue) for marker gene expression in B cells. n=8 tumors, n=6 AM samples. **C.** UMAP plot, color-coded for the distribution pattern of tumor-derived and adjacent mucosa (AM)-derived B cells. n=8 tumors, n=6 AM samples. **D.** Heatmap showing the top 10 GO terms enriched in either tumor-derived naïve B cells or AM-derived naïve B cells. The color represents the p value (from gray to red). **E.** UMAP plot color-coded for the distribution pattern of tumor-derived and adjacent mucosa (AM)-derived macrophages n=8 tumors, n=7 AM samples. **F.** UMAP plot color-coded (gray to blue) for marker gene expression in macrophages. n=8 tumors, n=7 AM samples. **G.** UMAP plot of macrophages showing 12 clusters colored according to clusters. 'TAM' represents tumor-associated macrophages. 'MDSC' represents myeloid-derived suppressor cells. **H.** Heatmap showing GO terms enriched in tumor-derived macrophages or AM-derived macrophages; the color represents the p value (from gray to red).

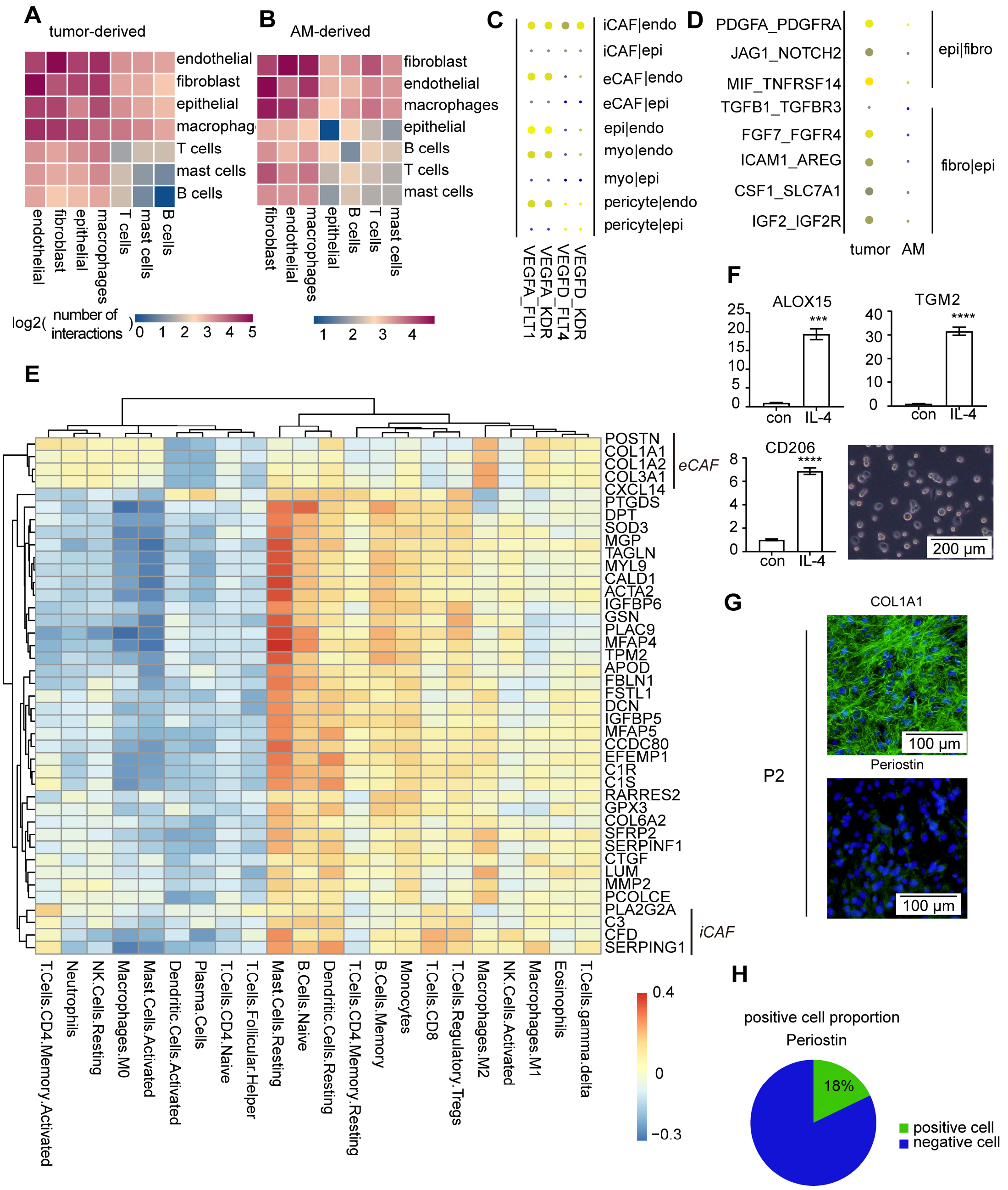

Figure S10: Communications among TME components. **A-B.** Heatmap showing interactions between seven cell types in tumor samples (**A**) or AM samples (**B**). The color represents the number of interactions (blue to red). n=8 tumors, n=8 AM samples. **C.** Selected specific interactions between CAF subgroups and epithelial cells (Epi) or endothelial cells (Endo) using the receptor-ligand pair analysis tool CellPhoneDB. Cells derived from the eight tumor samples were used for the analysis. The size indicates the

p value, and the color indicates the mean value of the receptor/ligand pairs between two clusters. **D.** Specific interactions between epithelial cells (Epi) and fibroblasts were selected. **E.** Heatmap showing the Spearman correlations between marker gene expression and the relative abundance of different immune cell types in TCGA cohort normalized to the fraction of stromal content. **F.** Bar plots showing the relative expression of *ALOX15* (P value = 0.0002), *TGM2* (P value <0.0001) and *CD206* (P value <0.0001). The p value was calculated using the unpaired t test. The photograph showing adherent Thp1 cells. **G.** Images of immunofluorescence staining in GC tumor-derived cells with antibodies against alpha-1 type 1 collagen (COL1A1) (green) and periostin (green). Scale bar, 100 μm. **H.** Pie chart showing the proportion of periostin-positive cells in the second generation of CAFs, as calculated using ImageJ software.
